# Totally Endoscopic Approach for Aortic Valve Replacement: A Systematic Review and Single-Arm Meta-Analysis

**DOI:** 10.3390/medicina62020339

**Published:** 2026-02-07

**Authors:** Florin Anghel, Mircea Ioan Alexandru Bistriceanu, Cristian Valentin Toma, Cosmin Gabriel Ursu, Andrei Dăneț, Andreea Dana Carolin Blindaru, Maria-Alis Popescu, Maria-Andrada Păun, Vlad-Ionuț Pârsan, Teodora Cornelia Mărgineanu, Daria Ileana Cristea, Cristiana Flavia Cristea, Alexia-Maria Ceaușu, Roxana Andreea Boboruță, Victoria-Nicoleta Alexandra Udrea, Darie Ioan Andreescu, Cătălin-Constantin Badiu

**Affiliations:** 1 Department of Cardiovascular Surgery, Emergency University Hospital of Bucharest, 050098 Bucharest, Romania; florin.anghel@rez.umfcd.ro (F.A.);; 2 Faculty of Medicine, Carol Davila University of Medicine and Pharmacy, 050474 Bucharest, Romania; cosmin-gabriel.ursu25@rez.umfcd.ro (C.G.U.); maria-andrada.paun0721@stud.umfcd.ro (M.-A.P.); vlad-ionut.parsan0721@stud.umfcd.ro (V.-I.P.); teodora-cornelia.margineanu2022@stud.umfcd.ro (T.C.M.); daria-ileana.cristea0721@stud.umfcd.ro (D.I.C.); cristiana-flavia.cristea0721@stud.umfcd.ro (C.F.C.); roxana-andreea.boboruta0721@stud.umfcd.ro (R.A.B.); victoria-nicoleta-alexandra.udrea0721@stud.umfcd.ro (V.-N.A.U.); darie-ioan.andreescu0720@stud.umfcd.ro (D.I.A.); 3 Curtin Medical School, Curtin University, Perth 6845, Australia

**Keywords:** endoscopic cardiac surgery, minimally invasive cardiac surgery, endoscopic aortic valve replacement

## Abstract

*Background and Objectives*: Totally endoscopic aortic valve replacement (TE-AVR) is a minimally invasive technique offering potential benefits of reduced surgical trauma and faster recovery compared with median sternotomy or other minimally invasive access. While isolated aortic valve replacement (AVR) is well established through conventional and minimally invasive access, large-scale evidence for the totally endoscopic approach remains limited. This meta-analysis aimed to systematically assess the safety and feasibility of TE-AVR by aggregating perioperative outcomes, including mortality, stroke, conversion, bleeding, paravalvular leak (PVL), and atrial fibrillation (AF). *Materials and Methods*: A systematic search of PubMed, Embase, and the Cochrane Library was performed, following PRISMA 2020 guidelines. Observational studies and randomized controlled trials reporting outcomes of totally endoscopic or thoracoscopic AVR were eligible. After independent screening and selection, data were analyzed using a single-arm proportion model. Leave-one-out sensitivity analyses were performed to evaluate the influence of individual studies. The protocol was registered in PROSPERO (CRD42024610128). *Results*: A total of 11 studies comprising 1135 patients were included. The pooled perioperative mortality was 0.00% (95% CI 0.00–0.23; I^2^ = 0.0%), indicating highly consistent results across cohorts. The stroke incidence was 0.69% (95% CI 0.00–2.07; I^2^ = 42.7%), confirming the low cerebrovascular risk of this approach. Conversion to sternotomy occurred in 0.00% of cases (95% CI 0.00–0.17; I^2^ = 0.0%), with no statistical heterogeneity observed. Reintervention for bleeding occurred in 1.75% (95% CI 0.34–3.85; I^2^ = 43.4%), while PVL was reported in 1.24% (95% CI 0.00–4.22; I^2^ = 64.0%). AF incidence was 10.54% (95% CI 3.79–19.70; I^2^ = 90.5%), with substantial between-study heterogeneity, likely related to non-standardized definitions of new-onset AF and variability in postoperative rhythm monitoring and reporting across studies. *Conclusions*: TE-AVR is a safe and feasible technique associated with very low perioperative mortality, bleeding, and stroke rates, as well as low PVL incidence. The absent conversion rate in our pooled analysis highlights the technical reliability of the procedure. Variability in AF reporting underscores the need for future randomized studies with harmonized definitions. Overall, TE-AVR offers a promising minimally invasive alternative for aortic valve replacement, with potential advantages in recovery (pooled ICU stay 1.86 days), hospital stay (pooled 7.98 days), and aesthetic outcomes.

## 1. Introduction

Totally endoscopic aortic valve replacement (TE-AVR) is a minimally invasive technique offering at least non-inferior potential benefits of reduced surgical trauma and faster recovery compared with median sternotomy (MS) or other minimally invasive accesses [1,2,3]. While isolated aortic valve replacement (AVR) is well established through conventional and minimally invasive access, large-scale evidence for the totally endoscopic approach remains limited [1,4].

In the modern era, heart surgery is undergoing a paradigm shift toward a patient-centered model that emphasizes both durable therapeutic results and accelerated recovery [4,5]. Nowadays patients increasingly seek alternatives to the traditional median sternotomy for routine cardiac surgery procedures, as they are often hesitant to undergo the significant physical and psychological trauma associated with large thoracic incisions [4,6]. This evolution has turned patient satisfaction, cosmetic advantage, and early postoperative quality of life (QoL) into primary metrics for surgical success [5,7]. The rapid expansion of transcatheter aortic valve replacement (TAVR) has intensified the debate over the optimal management of low-risk and intermediate-risk patients, prompting surgeons to refine surgical techniques to remain competitive in terms of invasiveness and recovery speed [3,7]. Currently, minimally invasive aortic valve replacement (MIAVR), typically performed via partial mini-sternotomy, right anterior thoracotomy or transaxillary approach, is recognized as a safe and effective alternative to MS [1,2]. These techniques provide clinical outcomes, including mortality and morbidity, that are comparable to the gold standard while reducing blood transfusion requirements and hospital stays [2,4]. Despite these benefits, a point of controversy remains regarding the trade-off between the reduced trauma of smaller incisions and the potentially longer cardiopulmonary bypass and cross-clamp times, which some clinicians argue could negatively impact high-risk or frail populations [2,8].

The transition from direct-vision MIAVR to TE-AVR represents the next technological frontier [4]. By integrating high-definition 3D video endoscopy, automated suturing devices, and sutureless or rapid-deployment valves, surgeons can now perform complex valve replacements through port-like incisions as small as 2 cm [4,8,9]. This meta-analysis aimed to systematically assess the safety and feasibility of TE-AVR by aggregating perioperative outcomes, including mortality, stroke, conversion, bleeding, paravalvular leak (PVL), and atrial fibrillation (AF).

## 2. Materials and Methods

This systematic review and meta-analysis were conducted and reported in accordance with the Cochrane Collaboration Handbook for Systematic Review of Interventions and the Preferred Reporting Items for Systematic Reviews and Meta-Analysis (PRISMA) statement guidelines (Supplementary Table S1) [10]. The prospective meta-analysis protocol was registered on PROSPERO (CRD420250655280).

### 2.1. Search Strategy

We systematically searched PubMed, Embase, and the Cochrane Central Register of Controlled Trials from inception to November 2025 using the following search strategy: (“aortic valve replacement” OR AVR) AND (endoscopic OR thoracoscopic OR “minimally invasive”).

### 2.2. Study Population

Studies were eligible for inclusion if they met all of the following criteria: (1) randomized controlled trials or observational studies; (2) evaluated patients undergoing TE-AVR; and (3) included adult patients with aortic valve disease—namely aortic stenosis, aortic regurgitation, mixed aortic valve disease, or bicuspid aortic valve—requiring surgical AVR. Studies were additionally required to report the primary outcome and at least one of the predefined clinical endpoints of interest. Studies were excluded if they: (1) involved animal experiments; (2) did not include a TE-AVR cohort; (3) failed to report postoperative complication outcomes; (4) included patients undergoing other surgical or transcatheter approaches, such as mini-thoracotomy, mini-sternotomy, robotic-assisted surgery, TAVR, or conventional full sternotomy; or (5) did not report early mortality data.

### 2.3. Data Screening and Extraction

The reference lists of all included studies, as well as those of relevant systematic reviews and meta-analyses, were manually screened to identify additional eligible articles. In addition, three studies [11,12,13] that were included in a previously published network meta-analysis [14] were also retrieved through our database search, and their eligibility was confirmed. Study selection was independently performed by two reviewers (F.A. and M.I.A.B.), with full-text articles retrieved for all records considered potentially eligible by at least one reviewer. Full texts were subsequently assessed independently by the same reviewers, and any disagreements were resolved through discussion or, when necessary, adjudication by a third reviewer (C.C.B.). No automation tools were used during the study selection process. Data extraction was conducted independently by two reviewers (F.A. and M.I.A.B.) using a standardized Microsoft Excel form. When essential data were missing or unclear, corresponding authors were contacted by email, with one follow-up reminder sent after two weeks. No automation tools were used for data extraction. The following study- and patient-level variables were extracted when available: first author, year of publication, country of origin, study period, sample size, follow-up duration, mean age, sex distribution, baseline comorbidities, New York Heart Association (NYHA) functional class, echocardiographic characteristics, and type of implanted aortic valve.

### 2.4. Outcomes

The primary objective of this meta-analysis was to evaluate in-hospital mortality following TE-AVR, which was considered the primary endpoint reflecting early procedural safety and success. Secondary endpoints included: (I) early mortality within 30 days, (II) operative and postoperative time-related outcomes, including cardiopulmonary bypass (CPB) time, aortic cross-clamp (ACC) time, intensive care unit (ICU) stay, and total length of hospitalization, and (III) early postoperative complications, defined as events occurring during hospitalization or within the first 30 postoperative days. These complications comprised acute kidney injury, complete atrioventricular block, permanent pacemaker implantation (PPI), new-onset AF, stroke, PVL, conversion to conventional surgery, and respiratory complications.

### 2.5. Risk of Bias

We assessed the risk of bias using the Risk of Bias in Non-Randomised Studies of Interventions, version 2 (ROBINS-I V2) assessment tool [15]. Two independent authors carried out the risk of bias assessment (F.A. and M.I.A.B.). Any disagreements were resolved through consensus after discussing the reasons for the discrepancy.

### 2.6. Statistical Analysis

Meta-analyses were conducted using random-effects models to account for both within-study sampling error and between-study heterogeneity. This approach was chosen a priori, given the expected clinical and methodological variability across the included observational studies. For continuous outcomes, pooled estimates were calculated using untransformed (raw) means, applying the inverse-variance method under a random-effects framework. Between-study variance (τ^2^) was estimated using the restricted maximum-likelihood (REML) estimator, which provides unbiased and efficient estimates of heterogeneity, particularly in meta-analyses with a limited number of studies. Confidence intervals for τ^2^ and τ were derived using the Q-profile method. Statistical heterogeneity was assessed using Cochran’s Q statistic and quantified using the I^2^ statistic, calculated based on the Q distribution, with higher values indicating a greater proportion of total variability attributable to between-study heterogeneity rather than chance. For binary outcomes, a single-arm meta-analysis of proportions was performed using a random-effects model. Event rates were pooled after applying the Freeman-Tukey double arcsine transformation to stabilize variances across the full range of proportions, including rare events and studies with zero events. Between-study variance was estimated using the REML method. Pooled estimates and corresponding 95% confidence intervals were back-transformed to the original proportion scale for interpretability. For all analyses, statistical heterogeneity was evaluated using Cochran’s Q test, with a significance threshold of *p* < 0.10 due to the limited power of the test when the number of included studies is small. Leave-one-out sensitivity analyses were conducted for outcomes with substantial heterogeneity (I^2^ ≥ 75%), and prespecified age-based subgroup analyses (<65 vs. ≥65 years) were performed to explore sources of heterogeneity.

To evaluate the contribution of individual studies to between-study heterogeneity and their influence on the pooled effect estimates, Baujat plots were generated using the *meta* package in R. These plots display each study according to its contribution to the overall heterogeneity on the x-axis and its influence on the pooled effect size on the y-axis, allowing identification of studies exerting a disproportionate impact on the meta-analytic results. Baujat plots were constructed for the primary outcome (in-hospital mortality) and for secondary outcomes exhibiting substantial heterogeneity (I^2^ ≥ 75%), to explore potential sources of between-study variability. Potential small-study effects and publication bias were assessed using funnel plots, in which effect estimates were plotted against their standard errors. In the absence of small-study effects, studies are expected to be symmetrically distributed around the pooled effect estimate, forming an inverted funnel shape. For outcomes with at least 10 contributing studies, funnel plot asymmetry was formally evaluated using Egger’s regression test. This test assesses the association between standardized effect estimates and their standard errors, with a statistically significant intercept suggesting the presence of small-study effects. Due to limited statistical power, formal tests for funnel plot asymmetry were not performed for outcomes with fewer than 10 studies. R version 4.5.0 (R Foundation for Statistical Computing, Vienna, Austria) was utilized for all statistical analyses.

## 3. Results

### 3.1. Study Selection

**Figure 1:**
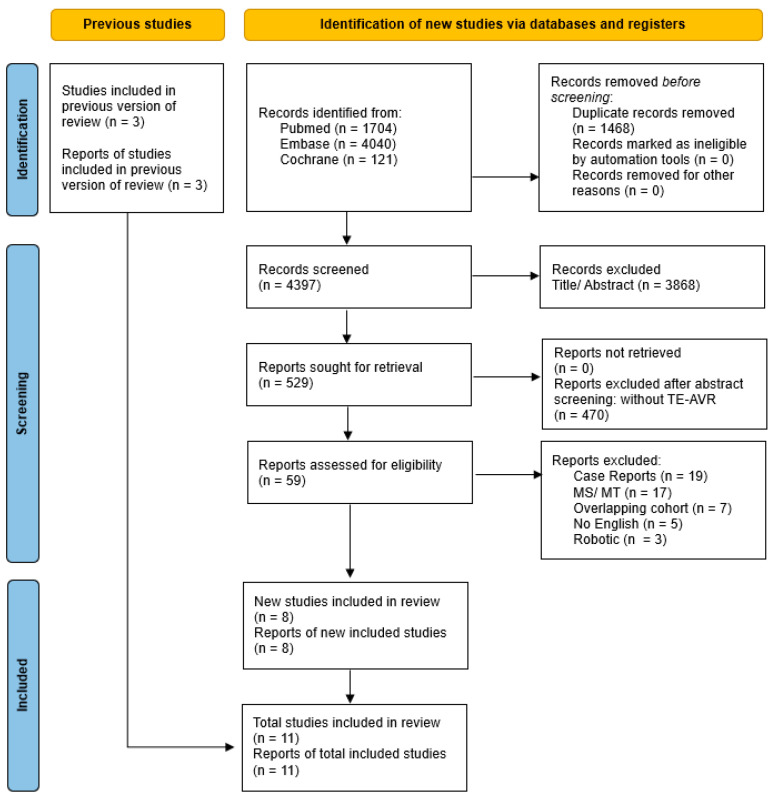
PRISMA flow chart [10].

Following the database search and manual screening of reference lists, a total of 4397 records were identified. After removal of duplicates and screening of titles and abstracts, 59 full-text articles were assessed for eligibility. Of these, 48 studies were excluded for predefined reasons, including the absence of a TE-AVR cohort, use of alternative surgical or transcatheter approaches, overlapping populations, non-English language, or lack of relevant outcome reporting. Eleven studies met the inclusion criteria and were included in the final quantitative synthesis. The study selection process is summarized in the PRISMA flow diagram (Figure 1).

### 3.2. Baseline Characteristics of Included Studies and Patients

The baseline characteristics of the included studies are presented in Table 1. All studies were published between 2019 and 2024 and originated predominantly from Asia and Europe, yielding a total of 1135 patients undergoing TE-AVR. Reported follow-up durations varied across studies; the sample-size weighted median follow-up was 15.3 months.

Baseline patient characteristics are summarized in Table 2. The pooled cohort had a mean age of 66.9 ± 13.3 years, and 39.1% of patients were female. Hypertension was the most frequently reported comorbidity (57.4%), followed by chronic kidney disease (28.4%) and diabetes mellitus (16.6%). Most patients were symptomatic, with approximately 60% classified as NYHA functional class II–III. From an echocardiographic perspective, aortic stenosis was the predominant indication for surgery (62.2%), followed by aortic regurgitation (26.9%) and mixed aortic valve disease (12.6%); 15.2% of patients had a bicuspid aortic valve. The majority of implanted prostheses were bioprosthetic valves (83.5%), whereas 16.4% were mechanical.

Extended baseline details are reported in Supplementary Table S2.

### 3.3. Primary Outcome Analysis

**Figure 2:**
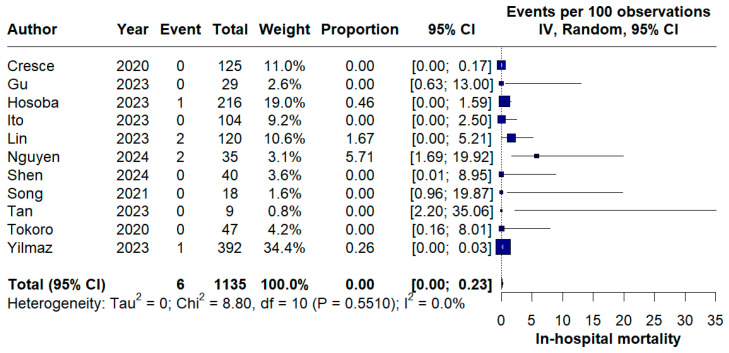
Forest plot of pooled in-hospital mortality after TE-AVR [11,12,13,16,17,18,19,20,21,22,23].

Across the 11 included studies, comprising a total of 1135 patients undergoing TE-AVR, in-hospital mortality was rare. A total of 6 in-hospital deaths were reported. The pooled in-hospital mortality rate was 0.0% (95% CI 0.0–0.23%) using a random-effects model. There was no evidence of between-study heterogeneity (I^2^ = 0.0%; *p* = 0.55), indicating highly consistent results across studies (Figure 2).

### 3.4. Procedural and Hospitalization-Related Endpoints

**Figure 3:**
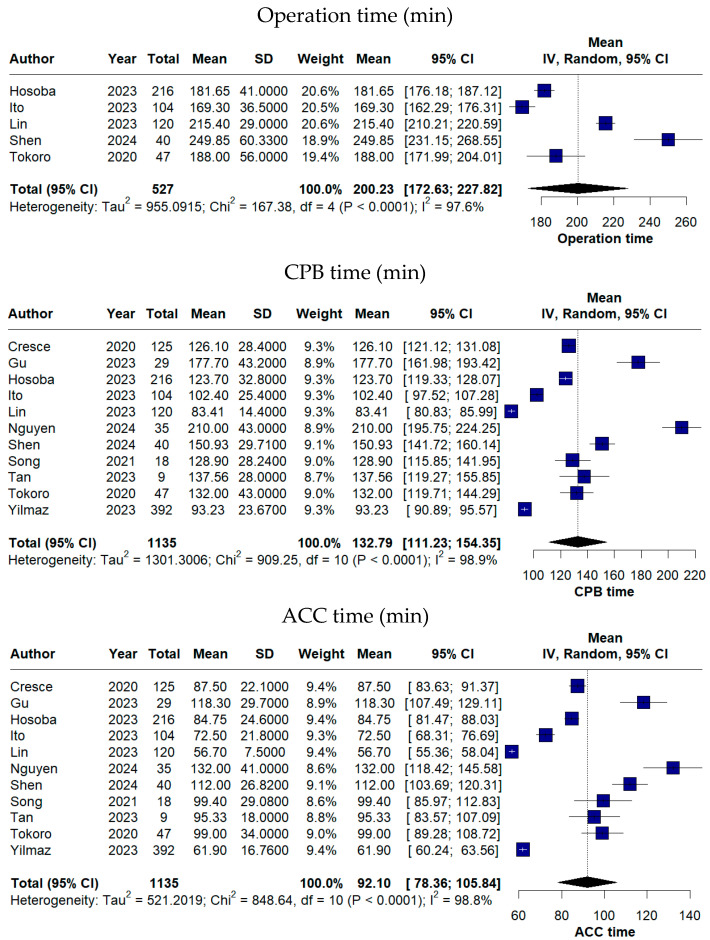
Forest plots of procedural time-related outcomes following TE-AVR (Operation time, cardiopulmonary bypass time and aortic cross-clamp time) [11,12,13,16,17,18,19,20,21,22,23].

A summary of procedural and hospitalization-related endpoints is provided in Table 3. Across the included studies, procedural and postoperative time-related outcomes demonstrated substantial variability, reflecting differences in surgical complexity, patient characteristics, and institutional experience. The pooled operation time was 200.23 min (95% CI 172.63–227.82), while the mean CPB time was 132.79 min (95% CI 111.23–154.35). The pooled ACC time was 92.10 min (95% CI: 78.36–105.84) (Figure 3).

**Figure 4:**
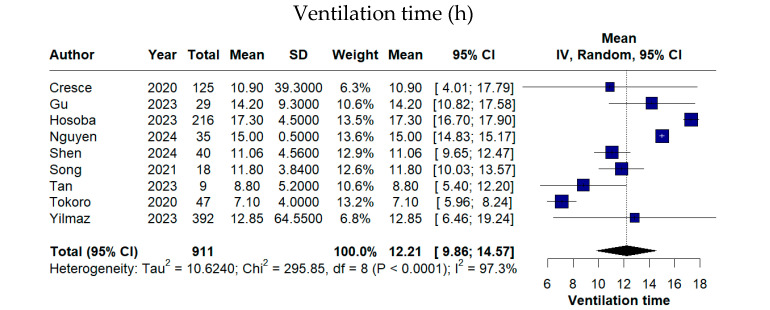

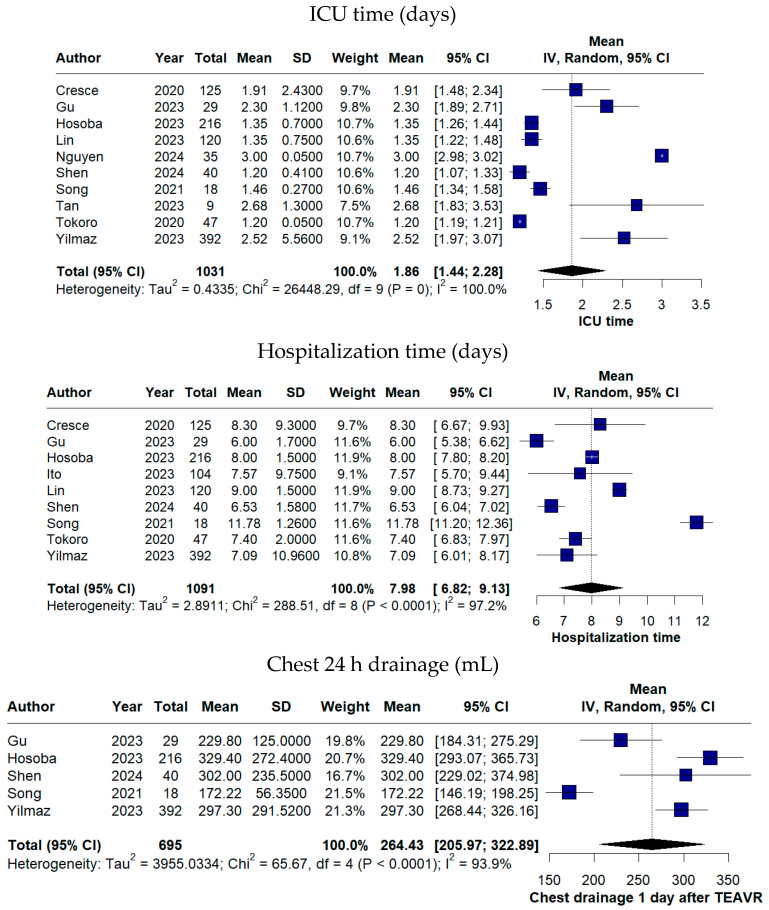
Forest plots of postoperative recovery and hospitalization-related outcomes following TE-AVR (ventilation time, intensive care unit stay, total hospitalization duration, and 24 h postoperative chest drainage) [11,12,13,16,17,18,19,20,21,22,23].

Postoperative recovery metrics showed a pooled ventilation time of 11.77 h (95% CI 9.21–14.34), an ICU stay of 1.86 days (95% CI 1.44–2.28), and a hospitalization duration of 7.98 days (95% CI 6.82–9.13). The pooled 24 h postoperative chest drainage volume was 264.43 mL (95% CI 205.97–322.89) (Figure 4). All time-related outcomes exhibited high between-study heterogeneity (I^2^ > 90% for all analyses), justifying the use of random-effects models and indicating considerable inter-study variability.

### 3.5. Early Postoperative Complications Following TE-AVR

A summary of early endpoints is provided in Table 4.

**Figure 5:**
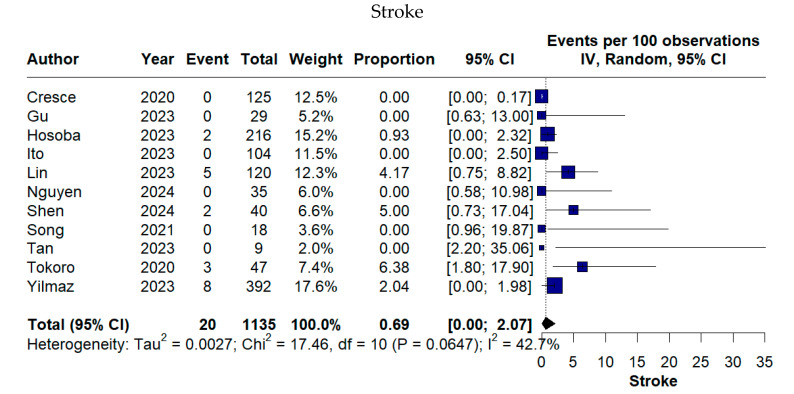

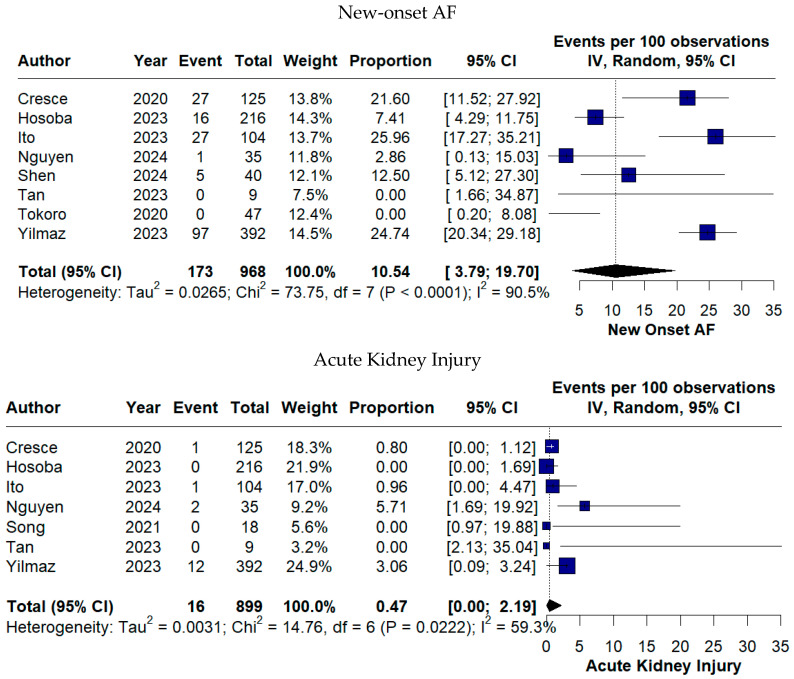
Forest plots of stroke, new-onset atrial fibrillation, and acute kidney injury after TE-AVR [11,12,13,16,17,18,19,20,21,22,23].

Across the included studies, early postoperative complications following TE-AVR were generally infrequent, although variability was observed across outcomes. The pooled incidence of stroke was 0.69% (95% CI 0.00–2.07%), while acute kidney injury occurred in 0.47% of patients (95% CI 0.00–2.19%). New-onset AF represented the most common cardiac complication, with a pooled rate of 10.54% (95% CI 3.79–19.70%) (Figure 5).

**Figure 6:**
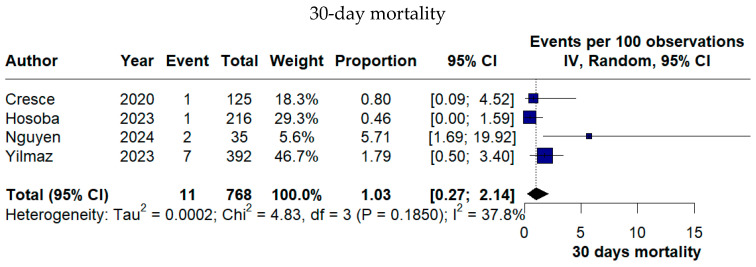

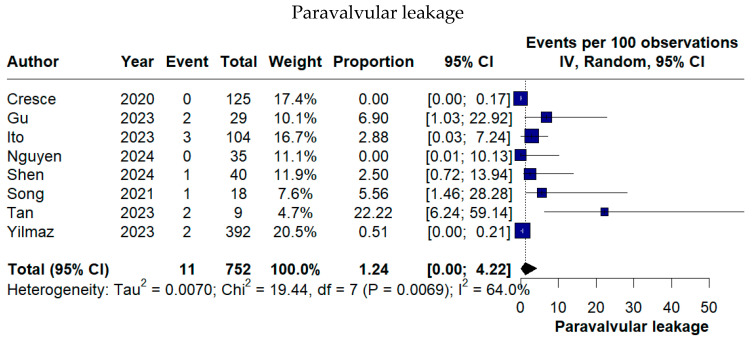
Forest plots of early mortality and valve-related complications following TE-AVR [11,12,13,16,17,18,19,22,23].

Early mortality remained low, with a pooled 30-day mortality rate of 1.03% (95% CI 0.27–2.14%). Valve-related complications were rare, as reflected by a pooled PVL rate of 1.24% (95% CI 0.00–4.21%) (Figure 6).

**Figure 7:**
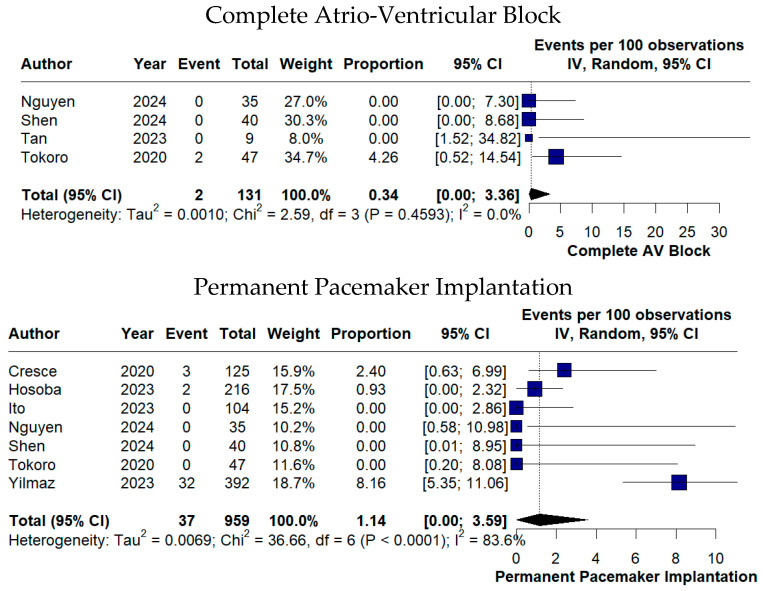
Forest plots of conduction-related complications following TE-AVR [11,12,16,17,18,20,22,23].

Conduction disturbances were uncommon, with complete atrioventricular block occurring in 0.34% of cases (95% CI 0.00–3.36%) and PPI required in 1.14% of patients (95% CI 0.00–3.59%) (Figure 7).

**Figure 8:**
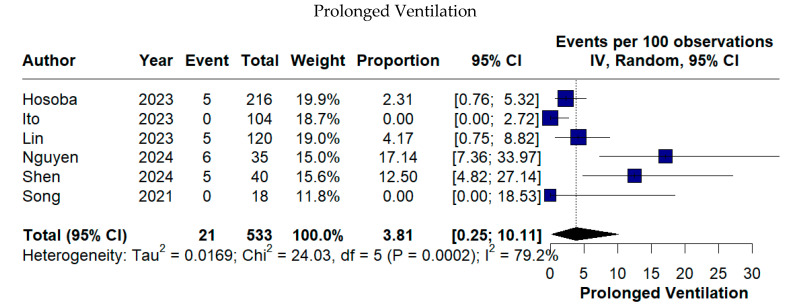

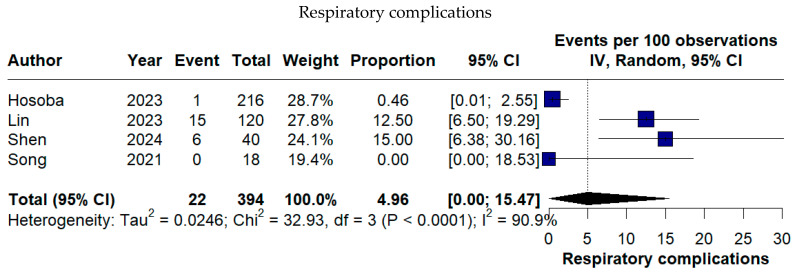
Forest plots of prolonged ventilation and postoperative respiratory complications following TE-AVR [12,13,17,18,21,23].

Respiratory morbidity was observed in a minority of cases, with prolonged ventilation occurring in 3.81% (95% CI 0.25–10.11%) and respiratory complications in 4.96% of patients (95% CI 0.00–15.47%) (Figure 8).

**Figure 9:**
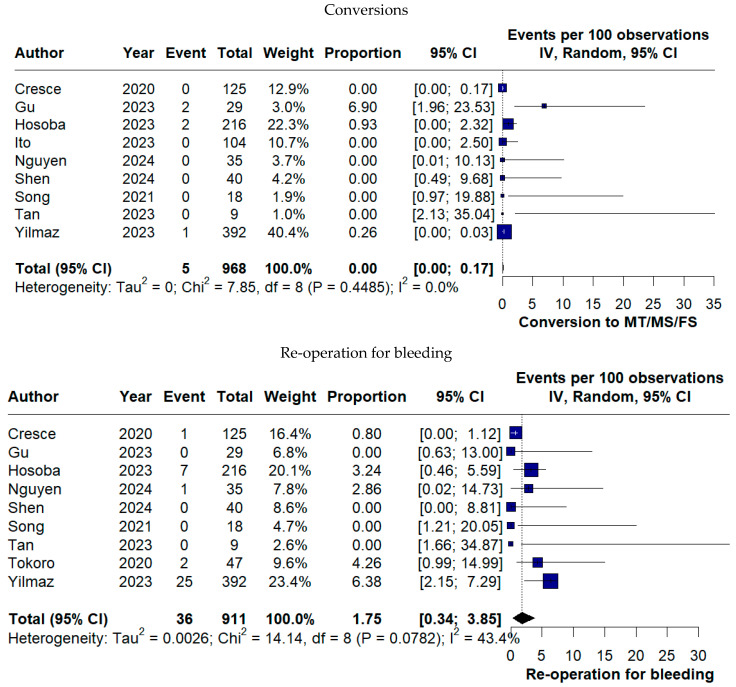

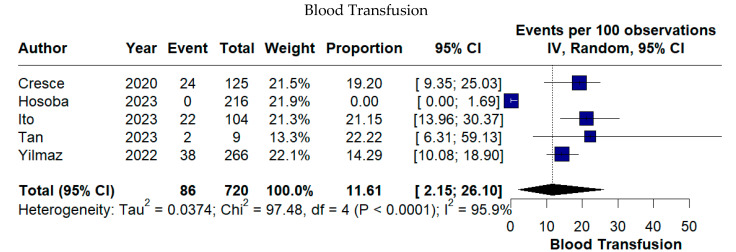
Forest plots of surgical conversion and bleeding-related complications following TE-AVR [11,12,13,16,17,18,19,20,22,23].

The need for conversion to alternative surgical approaches was exceptional (0.00%, 95% CI 0.00–0.17%). Bleeding-related outcomes included a pooled re-operation for bleeding rate of 1.75% (95% CI 0.34–3.85%), while blood transfusion was required in 11.61% of patients (95% CI 2.15–26.10%) (Figure 9).

Overall, these findings indicate a low incidence of major early complications after TEAVR, with substantial inter-study heterogeneity for several secondary endpoints.

### 3.6. Sensitivity Analyses

#### 3.6.1. Sensitivity Analyses of Early Postoperative Complications

A leave-one-out sensitivity analysis was performed for early postoperative complications to evaluate the robustness of pooled estimates and to explore sources of substantial between-study heterogeneity. Procedural and in-hospital time-related outcomes were not subjected to sensitivity analyses, as high heterogeneity was anticipated due to inherent differences in surgical complexity, institutional protocols, and perioperative management.

For PPI, the initial pooled incidence was 1.14% (95% CI 0.00–3.59; I^2^ = 83.6%). Exclusion of the study by Yilmaz et al. [11], which carried the highest weight and reported a higher event rate, resulted in a reduced pooled incidence of 0.42% (95% CI 0.00–1.37) and complete resolution of heterogeneity (I^2^ = 0%), indicating a strong influence of this study on between-study variability (Figure S1).

Similarly, for blood transfusion, the overall pooled incidence was 11.61% (95% CI 2.15–26.10) with substantial heterogeneity (I^2^ = 95.9%). After exclusion of the Hosoba et al. study [17], heterogeneity markedly decreased (I^2^ = 14.5%), while the pooled estimate remained comparable at 16.78% (95% CI 12.47–21.53), suggesting that heterogeneity was primarily driven by differences in transfusion practices rather than effect direction (Figure S2).

In the analysis of prolonged ventilation, the full model yielded a pooled incidence of 3.81% (95% CI 0.25–10.11; I^2^ = 79.2%). Sequential exclusion of influential studies demonstrated progressive reductions in heterogeneity. Removal of Ito et al. [23] reduced I^2^ to 56.9% (pooled incidence 3.54%, 95% CI 0.59–8.15), while further exclusion of Nguyen et al. [18] and Shen et al. [12] resulted in complete elimination of heterogeneity (I^2^ = 0%), with a pooled incidence of 2.10% (95% CI 0.63–4.14) (Figure S3). Importantly, pooled estimates remained within overlapping confidence intervals across all models.

For respiratory complications, the initial pooled incidence was 4.96% (95% CI 0.00–15.47) with high heterogeneity (I^2^ = 90.9%). Exclusion of Hosoba et al. [17] reduced heterogeneity to moderate levels (I^2^ = 57.9%) and yielded a pooled incidence of 9.00% (95% CI 2.08–19.26). Further exclusion of Song et al. [13] resulted in complete resolution of heterogeneity (I^2^ = 0%) with a pooled incidence of 12.95% (95% CI 8.04–18.74) (Figure S4).

Overall, leave-one-out sensitivity analyses demonstrated that heterogeneity in early complication outcomes was largely driven by a small number of influential studies, while pooled estimates remained directionally consistent, supporting the robustness of the primary findings.

#### 3.6.2. Subgroup Analysis by Mean Age

Age-stratified analyses (<65 vs. ≥65 years) showed uniformly low hospital mortality (<1%) and 30-day mortality (~1%) across subgroups (Figures S5 and S6). Rates of stroke, acute kidney injury, complete AV block, PPI, PVL, re-operation for bleeding, and conversions were comparable between age groups (Figures S7–S14). New-onset AF was more frequent in patients aged ≥65 years (13.8% vs. 5.1%) (Figure S9). Procedural and hospitalization times were highly heterogeneous in both subgroups (Figures S15–S17). No consistent age-related differences in early outcomes were identified.

#### 3.6.3. Exploration of Heterogeneity

Baujat plots for outcomes with high heterogeneity (I^2^ >75%) showed that overall heterogeneity was mainly driven by a limited number of studies, varying across endpoints (Figures S18–S30). In particular, Yilmaz [11], Nguyen [18], Song [13], Tokoro [20], Hosoba [17], and Lin [21] contributed disproportionately to heterogeneity, occasionally also exerting a higher influence on pooled estimates, while most remaining studies had minimal impact. These findings support the robustness of the results and justify the use of sensitivity and subgroup analyses.

### 3.7. Publication Bias

**Figure 10:**
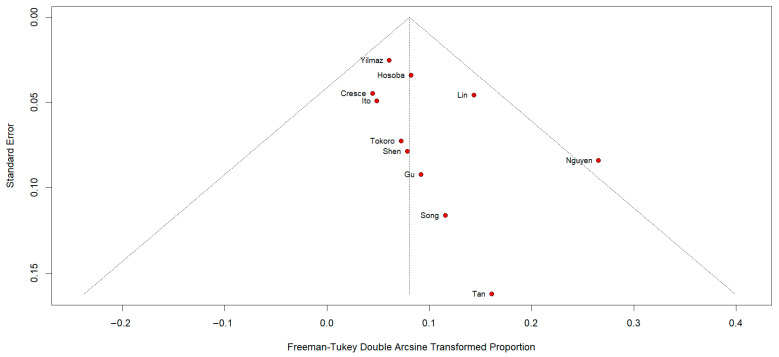
Funnel plot of hospital death [11,12,13,16,17,18,19,20,21,22,23].

Funnel plot inspection and Egger’s test did not reveal evidence of publication bias for hospital mortality (Figure 10), stroke, or ICU length of stay (Figures S31, S32 and S34). In contrast, CPB time showed significant funnel plot asymmetry, confirmed by Egger’s test (intercept 12.68, *p* = 0.002), suggesting the presence of small-study effects (Figure S33).

### 3.8. Risk of Bias Assessment

**Figure 11:**
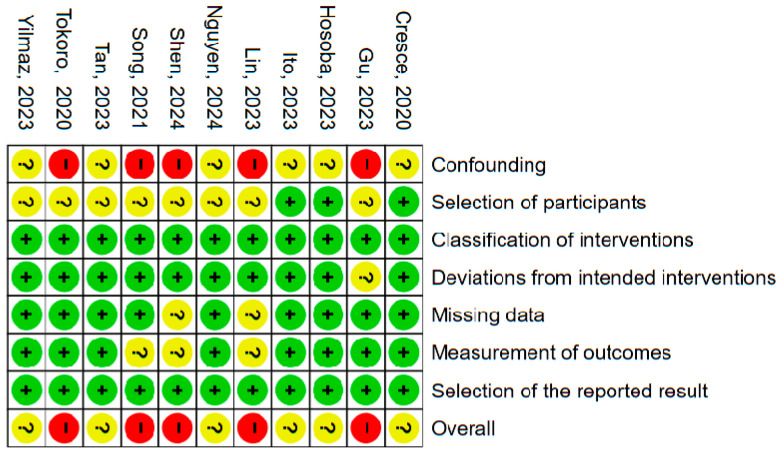
Risk of Bias Assessment of included studies [11,12,13,16,17,18,19,20,21,22,23].

The risk of bias of the included observational studies was evaluated using the ROBINS-I tool across seven domains: confounding, selection of participants, classification of interventions, deviations from intended interventions, missing data, measurement of outcomes, and selection of the reported result [15]. Overall, most studies were judged to have a low to moderate risk of bias. The domains related to the classification of interventions, deviations from intended interventions, and selection of reported outcomes were consistently assessed as low risk across the majority of studies. In contrast, confounding and selection of participants represented the main sources of concern, with several studies rated as having unclear or high risk due to the observational design, limited adjustment for baseline differences, or incomplete reporting of selection criteria. Missing data and outcome measurement were generally assessed as low or unclear risk, reflecting acceptable follow-up completeness and standardized outcome definitions. At the study level, the overall risk of bias was predominantly rated as moderate, with no study judged to be at critical risk. A detailed domain-level and study-level assessment is presented in Figure 11.

## 4. Discussion

The primary finding of this meta-analysis, encompassing a pooled cohort of 1135 patients, demonstrates that TE-AVR is a safe and technically feasible procedure characterized by an excellent perioperative safety profile, with an overall proportion of hospital death of 0%. The majority of individual studies reported zero events [12,13,16,19,20,22,23], with no statistical heterogeneity between studies. Compared with full-sternotomy and other minimally invasive approaches, such as mini-sternotomy, right anterior mini-thoracotomy or transaxillary approach, TE-AVR demonstrated at least non-inferior results [11,21]. In a recent network meta-analysis, Husen et al. found that short-term mortality was comparable across all minimally invasive techniques in a pooled cohort of 34,574 patients. Although the pooled mortality of our study is 0%, individual study differences observed in isolated single-centre studies showed that the learning curve, institutional protocols and patient selection are important factors influencing the outcomes [14].

TE-AVR demonstrates a low pooled incidence of stroke of 0.69%. This is clinically significant as the literature suggests that the totally endoscopic approach may offer better neurological protection compared to other minimally invasive approaches [14].

The most common complication observed is the new-onset of AF, with a pooled proportion of 10.54%. The high heterogeneity observed reflects different institutional protocols for postoperative monitoring and AF management, which is largely attributable to differences in outcome reporting across the included studies, without specification of diagnostic criteria, monitoring intensity, or temporal thresholds. This lack of standardized definition and ascertainment likely contributed to the wide dispersion of reported AF incidence and limits the interpretability of pooled estimates for this outcome. TE-AVR uses smaller incisions and avoids sternotomy, potentially reducing systemic inflammation and pericardial irritation, which are known triggers for AF. However, longer CPB and ACC times in TE-AVR could counterbalance this benefit [11]. Mini-thoracotomy and mini-sternotomy provide direct but limited access, while TE-AVR relies on endoscopic visualization and instrumentation, which may influence myocardial manipulation. TE-AVR is technically demanding with a steep learning curve, which might affect operative times and postoperative outcomes, including AF rates. High-volume centres with experienced surgeons report better outcomes [20]. The low PVL rate highlights the surgical advantage of direct vizualization for valve placement and decalcification. The low incidence of complete AV block and the pooled low rate for PPI are highly favorable compared to transcatheter procedures, which often reports double-digit PPI rates [7]. As surgical expertise continues to consolidate in the endoscopic field, TE-AVR could become a standard-of-care option for a broad range of patients, effectively bridging the gap between interventional cardiology and traditional cardiac surgery [3].

The conversion rate to mini-sternotomy or full sternotomy showed an overall proportion of 0%, indicating the technical reliability of the endoscopic approach. The pooled procedural times (operation time, CPB and ACC times) demonstrated very high heterogeneity, which is likely related to the learning curve associated with the advanced endoscopic procedures, where institutional experience also plays a major role [14]. TE-AVR is technically demanding and has been adopted at different stages of institutional experience across centres, with early series typically reporting longer operative and perfusion times. Variations in surgeon experience, case selection, and procedural standardization during the implementation phase of this technique likely contributed to the wide dispersion of CPB and ACC durations across studies. The implementation of new technologies, such as rapid-deployment valves and automated-suturing devices, represents potential solutions for the amelioration of operative times [4].

The clinical benefits of the TE-AVR approach are further reflected in the accelerated recovery of patients. Our results show a pooled ICU stay of 1.86 days and total hospitalization of 7.98 days. These metrics are vital for improving postoperative quality of life, as avoiding sternal trauma and rib spreading minimizes surgical pain and allows for faster physical rehabilitation [5,6]. Furthermore, the low rate of blood transfusion observed in our study (11.61%) highlights the reduced morbidity associated with this minimally invasive approach compared to more invasive conventional techniques. The technical success of TE-AVR has been bolstered by significant procedural innovations. The integration of 3D 4K endoscopic systems provides high-definition magnification, facilitating precise annular debridement and suture placement in the restricted “deep and narrow” operative field [8,12].

TE-AVR expanded to almost the entire spectrum of cardiovascular interventions. Recent studies show great promise in the field of aortic root surgery. Procedures such as root enlargement surgery or the Bentall procedure have been demonstrated to be safe and effective through a totally endoscopic approach [24,25]. The versatility of the endoscopic approach is further demonstrated by its use in complex reoperations, such as the explanation of failed TAVI prostheses [8]. As surgical expertise consolidates, TE-AVR is poised to become a definitive surgical alternative for all categories of risk patients seeking a balance between minimal invasiveness and long-term durability.

Despite these encouraging findings, the current evidence base remains limited by the absence of randomized controlled trials and by the predominantly short- to mid-term follow-up reported in the available studies. As such, the long-term durability, comparative effectiveness, and broader generalizability of TE-AVR will need to be confirmed in future randomized studies with extended follow-up.

### Limitations

This meta-analysis has several important limitations that should be acknowledged. First, the available evidence is derived exclusively from observational studies, predominantly single-center experiences, which inherently expose the results to selection bias, residual confounding, and center-specific practice patterns, despite the overall moderate risk of bias observed using the ROBINS-I tool. Second, the total number of included studies and patients remains limited, reflecting the early adoption phase of TE-AVR, and reducing the statistical power for several secondary endpoints, particularly rare complications. Third, substantial between-study heterogeneity was observed for multiple procedural and postoperative time-related outcomes, as well as for certain complications such as new-onset AF. Although sensitivity analyses, subgroup analyses, and Baujat plot exploration demonstrated that heterogeneity was largely driven by a small number of influential studies and that pooled estimates remained directionally consistent, this variability likely reflects differences in surgical expertise, learning curves, institutional protocols, patient selection, and reporting standards. Fourth, formal assessment of publication bias was limited by the small number of studies for several outcomes. While funnel plot inspection and Egger’s test did not suggest small-study effects for most endpoints, significant asymmetry was observed for CPB time, which may reflect clinical and methodological heterogeneity rather than publication bias alone. Finally, the absence of randomized controlled trials and direct comparative analyses with other minimally invasive or conventional surgical approaches precludes definitive conclusions regarding the superiority of TE-AVR. Long-term outcomes, including valve durability, late complications, and quality-of-life metrics, were inconsistently reported and could not be reliably synthesized. Future multicenter randomized studies with standardized definitions and longer follow-up are warranted to confirm these findings and better define the role of TE-AVR in contemporary aortic valve surgery.

## 5. Conclusions

Totally endoscopic aortic valve replacement is a safe and feasible procedure associated with very low perioperative mortality, low rates of major complications, short ICU stay, and a pooled length of eight days of hospitalization, supporting rapid postoperative recovery and potential cosmetic advantages. The consistency of results across included studies reinforces the feasibility of this approach in appropriately selected patients. The substantial heterogeneity observed for new-onset AF highlights the influence of institutional protocols, patient characteristics, and perioperative management strategies, underscoring the need for standardized reporting and further prospective studies.

## Figures and Tables

**Table 1:** Baseline characteristics of included studies [11,12,13,16,17,18,19,20,21,22,23].

Author	Year	Country	Study Period	Sample Size	Follow-Up (Months)
Tan [16]	2023	China	2021–2022	9	4.4 ± 2.7
Hosoba [17]	2023	Japan	2017–2022	216	15.3 ± 16.2
Nguyen [18]	2024	Vietnam	2023–2024	35	1
Gu [19]	2023	China	2020–2021	29	NA
Shen [12]	2024	China	NA	40	12
Yilmaz [11]	2023	Belgium	2013–2021	392	24 ± 17.1
Tokoro [20]	2020	Japan	2012–2018	47	1
Lin [21]	2023	China	2018–2022	120	12
Cresce [22]	2020	Italy	2013–2018	125	1
Ito [23]	2023	Japan	2015–2022	104	6
Song [13]	2021	China	2017–2021	18	12

NA = not available.

**Table 2:** Baseline characteristics of patients [11,12,13,16,17,18,19,20,21,22,23].

Variable	TE-AVR (*n* = 1135)
Age (years), mean ± SD	66.9 ± 13.3
Female, % (*n*)	39.1% (444/1135)
Comorbidities	
Smoking history, % (*n*)	25.2% (215/853)
Hypertension, % (*n*)	57.4% (635/1106)
Diabetus mellitus, % (*n*)	16.6% (189/1135)
Atrial fibrillation, % (*n*)	14.5% (155/1066)
Previous stroke, % (*n*)	5% (48/954)
Chronic kidney disease, % (*n*)	28.4% (312/1097)
COPD, % (*n*)	7% (50/716)
NYHA CLASS, % (*n*)	
● I	20.8% (208/1001)
● II	39.2% (392/1001)
● III	20.2% (214/1059)
● IV	7.9% (84/1059)
Echocardiographic details	
Baseline LVEF (%), mean ± SD	58.9 ± 19.8
Aortic stenosis, % (*n*)	62.2% (677/1088)
Aortic regurgitation, % (*n*)	26.9% (293/1088)
Mixed AS & AR, % (*n*)	12.6% (131/1041)
Bicuspid aortic valve, % (*n*)	15.2% (101/665)
Prosthesis type	
Mechanical, % (*n*)	16.4% (176/1070)
Bioprosthetic, % (*n*)	83.5% (893/1070)

COPD = chronic obstructive pulmonary disease, NYHA = New York Heart Association, LVEF = left ventricular ejection fraction, AS = aortic stenosis, AR = aortic regurgitation.

**Table 3:** Procedural and hospitalization-related endpoints [11,12,13,16,17,18,19,20,21,22,23].

Procedural Outcome	Pooled Mean	Total Size	95% CI
Operation time (min)	200.23	527	172.63–227.82
CPB time (min)	132.79	1135	111.23–154.35
ACC time (min)	92.10	1135	78.36–105.84
ICU time (days)	1.86	1031	1.44–2.28
Hospitalization time (days)	7.98	1091	6.82–9.13
Ventilation time (h)	11.77	911	9.21–14.34
Chest 24 h drainage (mL)	264.43	695	205.97–322.89

**Table 4:** Early complications after TE-AVR [11,12,13,16,17,18,19,20,21,22,23].

Complications	Events	Total Size	Proportion Result	95% CI
Hospital death	6	1135	0	0.00–0.23
30-day death	11	768	1.03	0.27–2.14
AKI	16	899	0.47	0.00–2.19
Complete AVB	2	131	0.34	0.00–3.36
PPI	37	959	1.14	0.00–3.59
New-onset AF	173	968	10.54	3.79–19.70
Stroke	20	1135	0.69	0.00–2.07
Paravalvular Leakage	11	752	1.24	0.00–4.22
Conversions	5	968	0	0.00–0.17
Respiratory Complications	22	394	4.96	0.00–15.47
Prolonged Ventilation	21	533	3.81	0.25–10.11
Re-operation (bleeding)	36	911	1.75	0.34–3.85
Blood Transfusion	86	720	11.61	2.15–26.10

AF—atrial fibrillation, AKI—acute kidney injury, AVB—atrio-ventricular block, PPI—permanent pacemaker implantation.

## Data Availability

This study is based on previously published data. No new data were generated or analyzed.

## Supplementary Materials

The following supporting information can be downloaded at: https://www.mdpi.com/article/10.3390/medicina62020339/s1, Table S1—PRISMA checklist, Table S2—Baseline characteristics of included studies and patients (extended table), Figures S1–S4—Leave-one-out analysis, Figures S5–S17—Subgroup analysis according to mean patient age (<65 vs. ≥65 years), Figures S18–S30—Baujat plot for outcomes with high heterogeneity (I^2^ > 75%), Figures S31–S34—Publication Bias (funnel plots and Eggers tests).

## Abbreviations

The following abbreviations are used in this manuscript:

ACC	Aortic cross-clamp
AF	Atrial fibrillation
AKI	Acute kidney injury
AR	Aortic regurgitation
AS	Aortic stenosis
AVR	Aortic valve replacement
AV	Atrio-ventricular
CI	Confidence interval
COPD	Chronic obstructive pulmonary disease
CPB	Cardiopulmonary bypass
ICU	Intensive care unit
I^2^	I-squared (heterogeneity statistic)
LVEF	Left ventricular ejection fraction
MIAVR	Minimally invasive aortic valve replacement
MS	Median sternotomy
MT	Mini-thoracotomy
NYHA	New York Heart Association
PPI	Permanent pacemaker implantation
PRISMA	Preferred Reporting Items for Systematic Reviews and Meta-analyses
PVL	Paravalvular leakage
QoL	Quality of life
REML	Restricted maximum likelihood
ROBINS-I	Risk of Bias in non-randomized studies of interventions
SD	Standard deviation
TE-AVR	Totally Endoscopic Aortic Valve Replacement
TAVR	Transcatheter Aortic Valve Replacement
