# Supplementary material for: Totally Endoscopic Approach for Aortic Valve Replacement: A Systematic Review and Single-Arm Meta-Analysis
Source: Medicina (Kaunas). 2026 Feb 7;62(2):339. doi: 10.3390/medicina62020339 (PMC12942395; doi:10.3390/medicina62020339)
Supplement: Supplementary file 1 [file medicina-62-00339-s001.zip › medicina-4113697-supplementary.pdf]

Section and Topic	Item #	Checklist item	Location where item is reported
**TITLE**			
Title	1	Identify the report as a systematic review.	Page1, Lines 2-3
**ABSTRACT**			
Abstract	2	See the PRISMA 2020 for Abstracts checklist.	Page 1, Lines 25-26
**INTRODUCTION**			
Rationale	3	Describe the rationale for the review in the context of existing knowledge.	Page 2, 73-75
Objectives	4	Provide an explicit statement of the objective(s) or question(s) the review addresses.	Page 2, Lines 75-77
**METHODS**			
Eligibility criteria	5	Specify the inclusion and exclusion criteria for the review and how studies were grouped for the syntheses.	Pages 2-3, Lines 89-97
Information sources	6	Specify all databases, registers, websites, organisations, reference lists and other sources searched or consulted to identify studies. Specify the date when each source was last searched or consulted.	Page 2, Lines 84-87, Page 3, Lines 100-103
Search strategy	7	Present the full search strategies for all databases, registers and websites, including any filters and limits used.	Page 2, Lines 84-87
Selection process	8	Specify the methods used to decide whether a study met the inclusion criteria of the review, including how many reviewers screened each record and each report retrieved, whether they worked independently, and if applicable, details of automation tools used in the process.	Pages 2-3, Lines 89-97, Lines 100-103
Data collection process	9	Specify the methods used to collect data from reports, including how many reviewers collected data from each report, whether they worked independently, any processes for obtaining or confirming data from study investigators, and if applicable, details of automation tools used in the process.	Page 3, Lines 104-111
Data items	10a	List and define all outcomes for which data were sought. Specify whether all results that were compatible with each outcome domain in each study were sought (e.g. for all measures, time points, analyses), and if not, the methods used to decide which results to collect.	Page 3, Lines 113-121
	10b	List and define all other variables for which data were sought (e.g. participant and intervention characteristics, funding sources). Describe any assumptions made about any missing or unclear information.	Page 3, Lines 106-107
Study risk of bias assessment	11	Specify the methods used to assess risk of bias in the included studies, including details of the tool(s) used, how many reviewers assessed each study and whether they worked independently, and if applicable, details of automation tools used in the process.	Page 3, Lines 123-126
Effect measures	12	Specify for each outcome the effect measure(s) (e.g. risk ratio, mean difference) used in the synthesis or presentation of results.	Page 3, Lines 129-143
Synthesis methods	13a	Describe the processes used to decide which studies were eligible for each synthesis (e.g. tabulating the study intervention characteristics and comparing against the planned groups for each synthesis (item #5)).	Page 3, Lines 100-111

Section and Topic	Item #	Checklist item	Location where item is reported
	13b	Describe any methods required to prepare the data for presentation or synthesis, such as handling of missing summary statistics, or data conversions.	Page 3, Lines 100-111
	13c	Describe any methods used to tabulate or visually display results of individual studies and syntheses.	Page 3, Lines 100-111
	13d	Describe any methods used to synthesize results and provide a rationale for the choice(s). If meta-analysis was performed, describe the model(s), method(s) to identify the presence and extent of statistical heterogeneity, and software package(s) used.	Page 3, Lines 129-143, Page 4,Lines 158-159
	13e	Describe any methods used to explore possible causes of heterogeneity among study results (e.g. subgroup analysis, meta-regression).	Page 4, Lines 146-159
	13f	Describe any sensitivity analyses conducted to assess robustness of the synthesized results.	Page 3, Lines 143-145
Reporting bias assessment	14	Describe any methods used to assess risk of bias due to missing results in a synthesis (arising from reporting biases).	Page 3, Lines 123-126
Certainty assessment	15	Describe any methods used to assess certainty (or confidence) in the body of evidence for an outcome.	Not applicable
**RESULTS**			
Study selection	16a	Describe the results of the search and selection process, from the number of records identified in the search to the number of studies included in the review, ideally using a flow diagram.	Page 4, Lines 162-169, Page 5
	16b	Cite studies that might appear to meet the inclusion criteria, but which were excluded, and explain why they were excluded.	Page 5
Study characteristics	17	Cite each included study and present its characteristics.	Page 4, Lines 171-184, Page 6
Risk of bias in studies	18	Present assessments of risk of bias for each included study.	Pages 16-17, Lines 452-466
Results of individual studies	19	For all outcomes, present, for each study: (a) summary statistics for each group (where appropriate) and (b) an effect estimate and its precision (e.g. confidence/credible interval), ideally using structured tables or plots.	Pages 7-14
Results of syntheses	20a	For each synthesis, briefly summarise the characteristics and risk of bias among contributing studies.	Pages 16-17, Lines 452-467
	20b	Present results of all statistical syntheses conducted. If meta-analysis was done, present for each the summary estimate and its precision (e.g. confidence/credible interval) and measures of statistical heterogeneity. If comparing groups, describe the direction of the effect.	Pages 7-14
	20c	Present results of all investigations of possible causes of heterogeneity among study results.	Page 16, Lines 437-443
	20d	Present results of all sensitivity analyses conducted to assess the robustness of the synthesized results.	Pages 15-16, Lines 398-436

Section and Topic	Item #	Checklist item	Location where item is reported
Reporting biases	21	Present assessments of risk of bias due to missing results (arising from reporting biases) for each synthesis assessed.	Page 16, Lines 444-450
Certainty of evidence	22	Present assessments of certainty (or confidence) in the body of evidence for each outcome assessed.	Not applicable
**DISCUSSION**			
Discussion	23a	Provide a general interpretation of the results in the context of other evidence.	Pages 17-18, Lines 468-505
	23b	Discuss any limitations of the evidence included in the review.	Page 18 Lines 513-533
	23c	Discuss any limitations of the review processes used.	Page 18, Lines 513-533
	23d	Discuss implications of the results for practice, policy, and future research.	Page 18, Lines 506-511
**OTHER INFORMATION**			
Registration and protocol	24a	Provide registration information for the review, including register name and registration number, or state that the review was not registered.	Page 2, Lines 81-82
	24b	Indicate where the review protocol can be accessed, or state that a protocol was not prepared.	Page 2, Lines 81-82
	24c	Describe and explain any amendments to information provided at registration or in the protocol.	Page 2, Lines 81-82
Support	25	Describe sources of financial or non-financial support for the review, and the role of the funders or sponsors in the review.	Page 19, Line 556
Competing interests	26	Declare any competing interests of review authors.	Page 19, Line 563
Availability of data, code and other materials	27	Report which of the following are publicly available and where they can be found: template data collection forms; data extracted from included studies; data used for all analyses; analytic code; any other materials used in the review.	Page 19, Lines 543-549

Table S1. PRISMA checklist [1]

Author	Year	Country	Study period	Sample size	Follow-up (months)	Female, n(%)	Age	Hypertension	Diabetes mellitus	AF	Stroke history	CKD	COPD	LVEF baseline	LVEDD baseline	NYHA I	NYHA II	NYHA III	NYHA IV	History of Smoking	AS	AR	AS + AR	Bicuspid AV	Mechanical valve	Bioprosthetic valve
Tan [2]	2023	China	2021 - 2022	9	4.4 ± 2.7	3	50.89 ± 5.30	1	0	0	NA	NA	NA	60.9 ± 9	NA	0	5	4	0	0	5	4	0	6	7	2
Hosoba [3]	2023	Japan	2017 - 2022	216	15.3 ± 16.2	102	71.3 ± 11.3	141	35	39	8	23	4	64.4 ± 12.2	52.2 ± 14.5	0	0	39	39	77	131	80	5	NA	22	194
Nguyen [4]	2024	Vietnam	2023 - 2024	35	1	8	58.7 ±12.8	15	5	3	0	0	1	53.1 ± 29.2	53.5 ± 10.9	6	23	5	1	NA	12	8	15	0	11	24
Gu [5]	2023	China	2020 - 2021	29	NA	9	51.7 ± 11.8	NA	0	NA	NA	NA	0	61.0 ± 7.9	58.8 ± 10.1	NA	NA	NA	NA	4	NA	NA	NA	NA	20	9
Shen [6]	2024	China	NA	40	12	7	51.67 ± 16	15	5	NA	1	1	0	61.2 ± 7.4	NA	NA	NA	4	4	14	9	22	9	NA	13	27
Yilmaz [7]	2023	Belgium	2013 - 2021	392	24 ± 17.1	158	71.20 ± 10.85	238	87	61	24	270	NA	58.8 ± 35.8	NA	124	220	43	4	78	357	17	18	72	7	384
Tokoro [8]	2020	Japan	2012 - 2018	47	1	30	71.7 ± 15	27	7	19	3	0	0	68.3 ± 12	NA	NA	NA	NA	NA	12	37	10	NA	NA	NA	NA
Lin [9]	20	China	2018 - 2022	120	12	53	56.3 ± 9	34	21	7	7	8	8	54 ± 6.4	NA	21	10	76	13	30	27	22	71	NA	96	24

	23																									
Cresce [10]	2020	Italy	2013 - 2018	125	1	49	68.8 ± 10.9	102	21	18	NA	4	24	58.5 ± 8.6	NA	19	74	27	5	NA	99	26	0	0	0	125
Ito [11]	2023	Japan	2015 - 2022	104	6	20	66.0 ± 14.3	57	6	6	5	6	13	53.3 ± 9.1	56.5 ± 8.4	38	60	6	0	NA	0	104	13	23	0	104
Song [12]	2021	China	2017 - 2021	18	12	5	53.83 ± 8.6	5	2	2	NA	0	NA	62.4 ± 7.2	56.7 ± 7.7	NA	NA	10	18	NA	NA	NA	NA	NA	NA	NA

Table S2. Baseline characteristics of included studies and patients [2–12].

AF = atrial fibrillation, AR = aortic regurgitation, AS = aortic stenosis, AV = aortic valve, CKD = Chronic kidney disease, COPD = chronic obstructive pulmonary disease, NA = not available, LVEDD = left ventricular end-diastole diameter, LVEF = left ventricular ejection fraction, NYHA = New York Heart Association

Author	Year	Event	Total	Weight	Proportion	95% CI
Cresce	2020	3	125	22.0%	2.40	[0.63; 6.99]
Hosoba	2023	2	216	38.0%	0.93	[0.00; 2.32]
Ito	2023	0	104	18.3%	0.00	[0.00; 2.86]
Nguyen	2024	0	35	6.2%	0.00	[0.05; 10.27]
Shen	2024	0	40	7.1%	0.00	[0.28; 9.46]
Tokoro	2020	0	47	8.3%	0.00	[0.16; 8.01]
**Total (95% CI)**		**5**	**567**	**100.0%**	**0.42**	**[0.00; 1.37]**

Heterogeneity: $Tau^2 = 0$; $Chi^2 = 3.62$, df = 5 (P = 0.6046); $I^2 = 0.0\%$

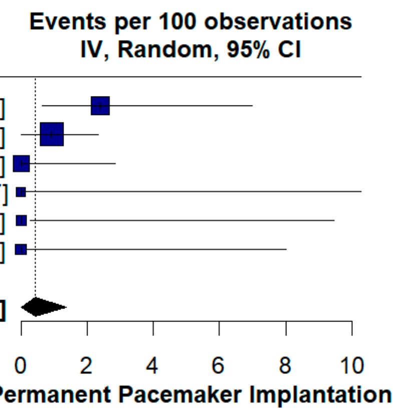

Figure S1. Leave-one-out sensitivity analysis for permanent pacemaker implantation, after exclusion of the study by Yilmaz et al. [7], showing the effect on pooled incidence and between-study heterogeneity [3,4,6,8,10,11].

Author	Year	Event	Total	Weight	Proportion	95% CI
Cresce	2020	24	125	28.4%	19.20	[9.35; 25.03]
Ito	2023	22	104	25.1%	21.15	[13.96; 30.37]
Tan	2023	2	9	3.2%	22.22	[6.31; 59.13]
Yilmaz	2022	38	266	43.4%	14.29	[10.08; 18.90]
**Total (95% CI)**		**86**	**504**	**100.0%**	**16.78**	**[12.47; 21.53]**

Heterogeneity: $Tau^2 = 0.0010$; $Chi^2 = 3.51$, df = 3 (P = 0.3194); $I^2 = 14.5\%$

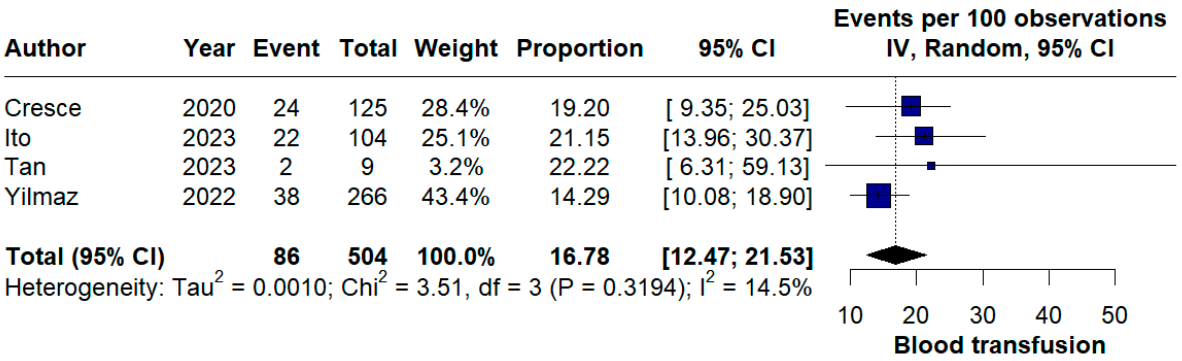

Figure S2. Leave-one-out sensitivity analysis for blood transfusion, after exclusion of the study by Hosoba et al. [3], assessing the robustness of the pooled estimate [2,7,10,11].

Excluding Ito

Author	Year	Event	Total	Weight	Proportion	95% CI
Hosoba	2023	5	216	26.0%	2.31	[0.76; 5.32]
Lin	2023	5	120	24.3%	4.17	[0.65; 8.68]
Nguyen	2024	6	35	17.8%	17.14	[6.70; 33.70]
Shen	2024	5	40	18.7%	12.50	[4.87; 27.17]
Song	2021	0	18	13.2%	0.00	[0.00; 18.53]
**Total (95% CI)**		**21**	**429**	**100.0%**	**5.45**	**[1.06; 12.20]**

Heterogeneity: $Tau^2 = 0.0116$; $Chi^2 = 14.60$, df = 4 (P = 0.0056); $I^2 = 72.6\%$

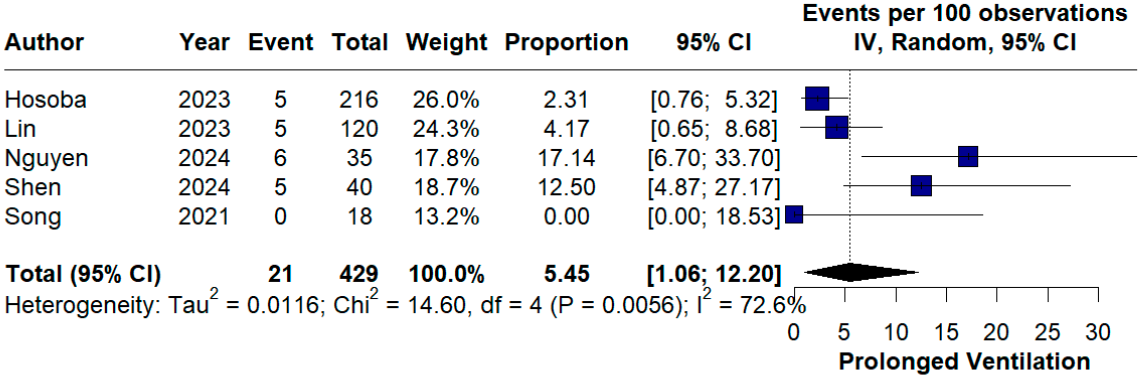

Excluding Ito and Nguyen

Author	Year	Event	Total	Weight	Proportion	95% CI
Hosoba	2023	5	216	36.6%	2.31	[0.76; 5.32]
Lin	2023	5	120	31.7%	4.17	[0.75; 8.82]
Shen	2024	5	40	19.8%	12.50	[4.87; 27.17]
Song	2021	0	18	11.9%	0.00	[0.00; 18.53]
**Total (95% CI)**		**15**	**394**	**100.0%**	**3.54**	**[0.59; 8.15]**

Heterogeneity: $Tau^2 = 0.0048$; $Chi^2 = 6.95$, df = 3 (P = 0.0734); $I^2 = 56.9\%$

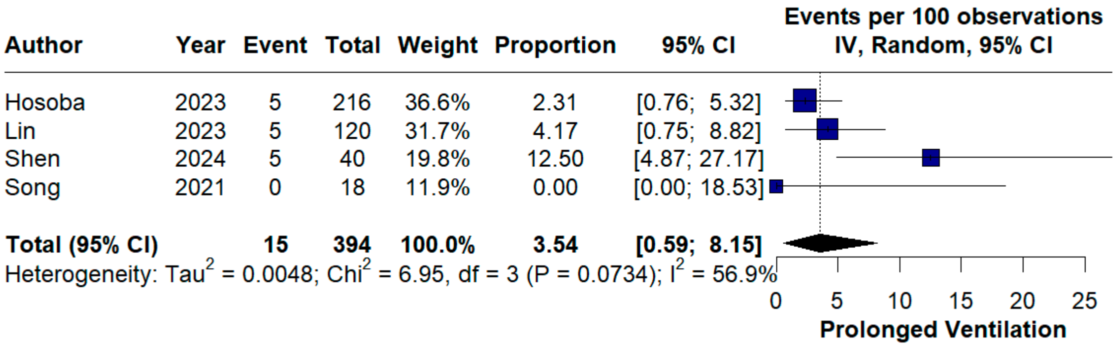

Excluding Ito, Nguyen and Shen

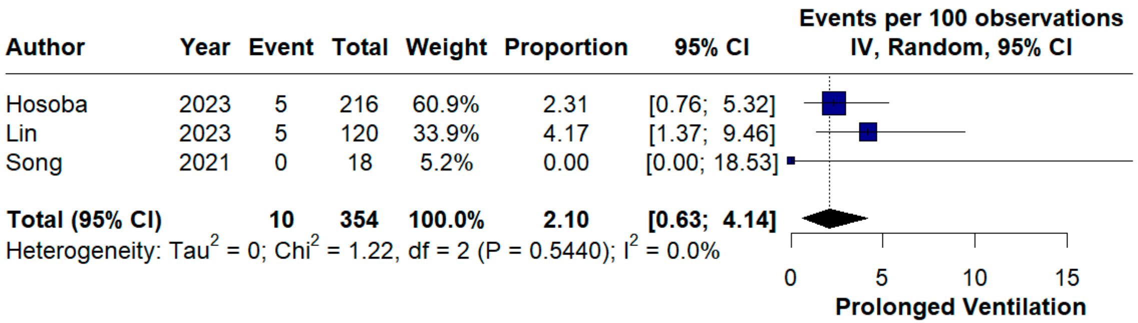

Figure S3.Leave-one-out sensitivity analysis for prolonged ventilation, after sequential exclusion of Ito et al. [11], Nguyen et al. [4], and Shen et al. [6], illustrating the impact of influential studies on heterogeneity [3,9,12].

Excluding Hosoba

Author	Year	Event	Total	Weight	Proportion	95% CI
Lin	2023	15	120	44.6%	12.50	[6.50; 19.29]
Shen	2024	6	40	33.0%	15.00	[6.38; 30.16]
Song	2021	0	18	22.5%	0.00	[0.00; 18.53]
**Total (95% CI)**		**21**	**178**	**100.0%**	**9.00**	**[2.08; 19.26]**

Heterogeneity: $Tau^2 = 0.0096$; $Chi^2 = 4.75$, df = 2 (P = 0.0929); $I^2 = 57.9\%$

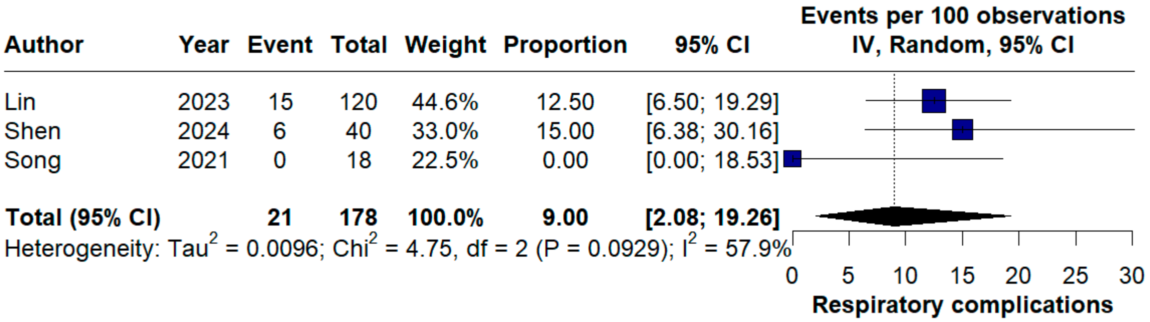

Excluding Hosoba and Song

Author	Year	Event	Total	Weight	Proportion	95% CI
Lin	2023	15	120	74.8%	12.50	[6.50; 19.29]
Shen	2024	6	40	25.2%	15.00	[6.38; 30.16]
**Total (95% CI)**		**21**	**160**	**100.0%**	**12.95**	**[8.04; 18.74]**

Heterogeneity: $Tau^2 = 0$; $Chi^2 = 0.23$, df = 1 (P = 0.6337); $I^2 = 0.0\%$

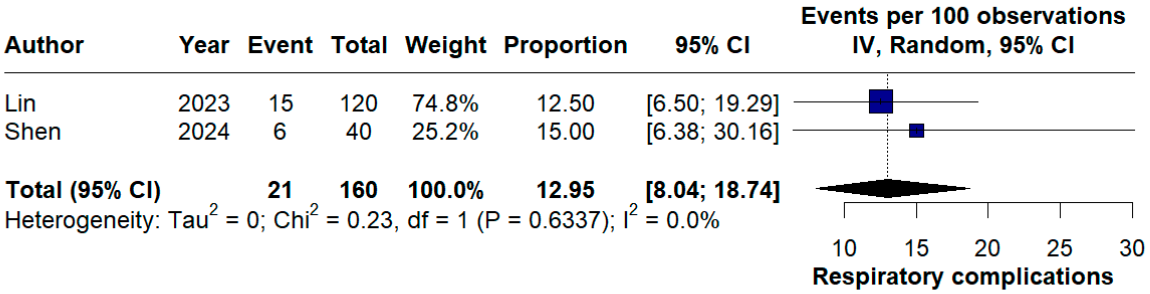

Figure S4. Leave-one-out sensitivity analysis for respiratory complications, after exclusion of Hosoba et al. [3], and subsequently Song et al. [12], evaluating changes in pooled incidence and heterogeneity [6,9].

< 65 years

Author	Year	Event	Total	Weight	Proportion	95% CI
Gu	2023	0	29	11.6%	0.00	[0.00; 9.53]
Lin	2023	2	120	47.4%	1.67	[0.00; 5.07]
Nguyen	2024	2	35	14.0%	5.71	[0.54; 18.99]
Shen	2024	0	40	15.9%	0.00	[0.00; 8.81]
Song	2021	0	18	7.3%	0.00	[0.96; 19.87]
Tan	2023	0	9	3.7%	0.00	[0.63; 34.38]
**Total (95% CI)**		**4**	**251**	**100.0%**	**0.48**	**[0.00; 2.37]**

Heterogeneity: $Tau^2 = 0$; $Chi^2 = 3.18$, df = 5 (P = 0.6723); $I^2 = 0.0\%$

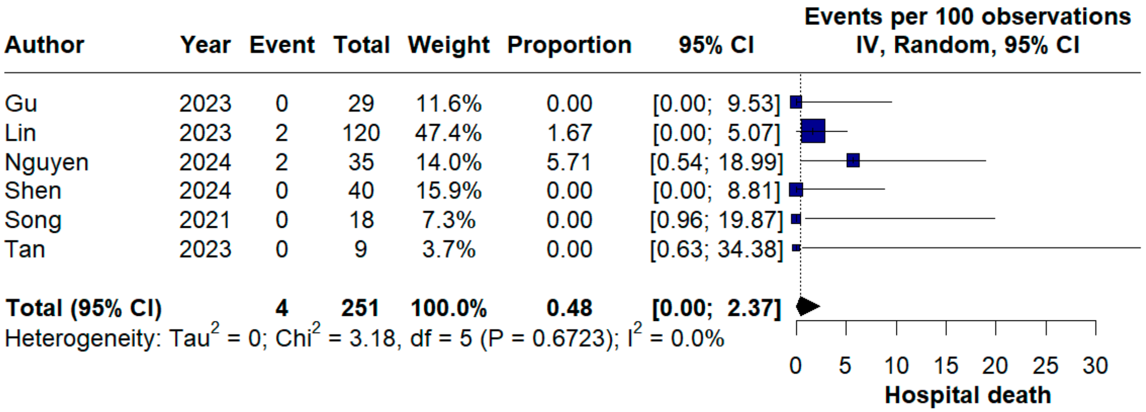

≥ 65 years

Author	Year	Event	Total	Weight	Proportion	95% CI
Cresce	2020	0	125	14.2%	0.00	[0.04; 3.06]
Hosoba	2023	1	216	24.4%	0.46	[0.06; 2.65]
Ito	2023	0	104	11.8%	0.00	[0.00; 3.00]
Tokoro	2020	0	47	5.4%	0.00	[0.20; 8.08]
Yilmaz	2023	1	392	44.3%	0.26	[0.00; 1.10]
**Total (95% CI)**		**2**	**884**	**100.0%**	**0.07**	**[0.00; 0.52]**

Heterogeneity: $Tau^2 = 0$; $Chi^2 = 0.59$, df = 4 (P = 0.9641); $I^2 = 0.0\%$

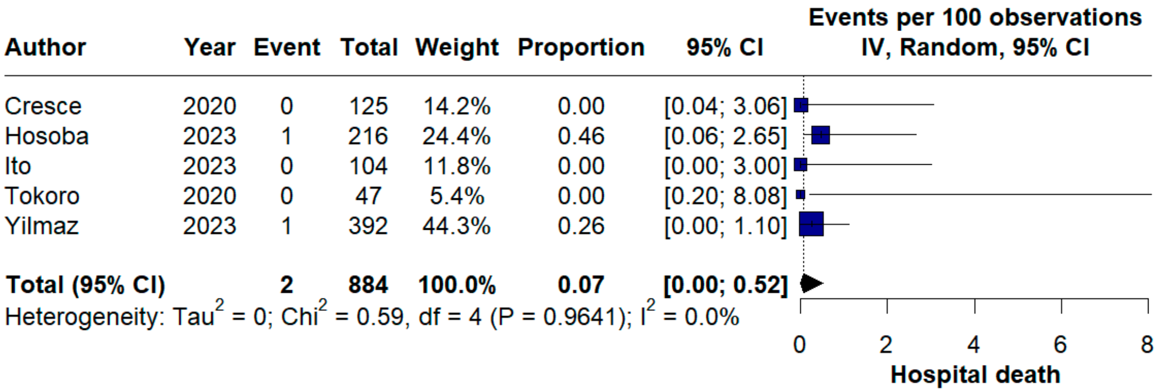

Figure S5. Subgroup analysis of in-hospital mortality after TE-AVR according to mean patient age < 65 years [2,4–6,9,12] vs ≥ 65 years [3,7,8,10,11].

Author	Year	Event	Total	Weight	Proportion	95% CI
Cresce	2020	1	125	18.6%	0.80	[0.09; 4.52]
Hosoba	2023	1	216	30.6%	0.46	[0.06; 2.65]
Yilmaz	2023	7	392	50.8%	1.79	[0.50; 3.40]
**Total (95% CI)**		**9**	**733**	**100.0%**	**1.09**	**[0.36; 2.14]**

Heterogeneity: $Tau^2 = 0.0001$; $Chi^2 = 1.83$, df = 2 (P = 0.4011); $I^2 = 0.0\%$

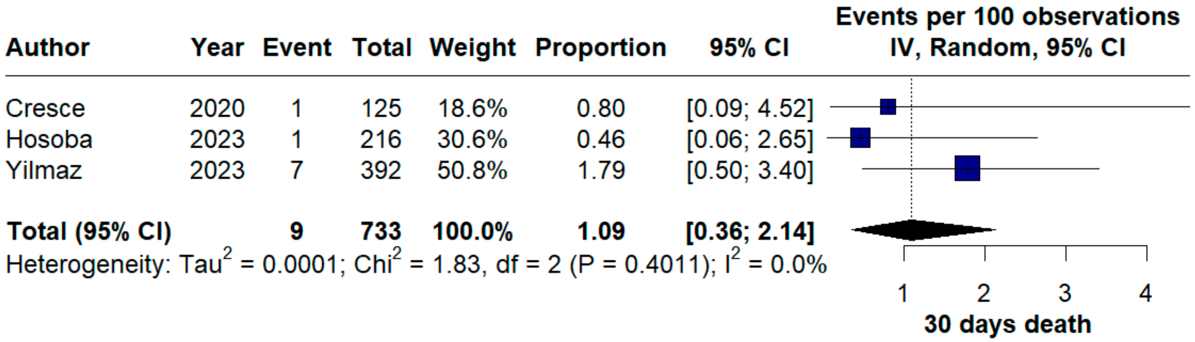

Figure S6.Subgroup analysis of 30-day mortality after TE-AVR in patients with a mean age ≥ 65 years [3,7,10].

Author	Year	Event	Total	Weight	Proportion	95% CI
Nguyen	2024	2	35	55.9%	5.71	[0.00; 16.92]
Song	2021	0	18	29.1%	0.00	[0.31; 19.29]
Tan	2023	0	9	15.0%	0.00	[0.63; 34.38]
**Total (95% CI)**		**2**	**62**	**100.0%**	**1.78**	**[0.00; 8.08]**

Heterogeneity: $Tau^2 = 0$; $Chi^2 = 1.18$, df = 2 (P = 0.5540); $I^2 = 0.0\%$

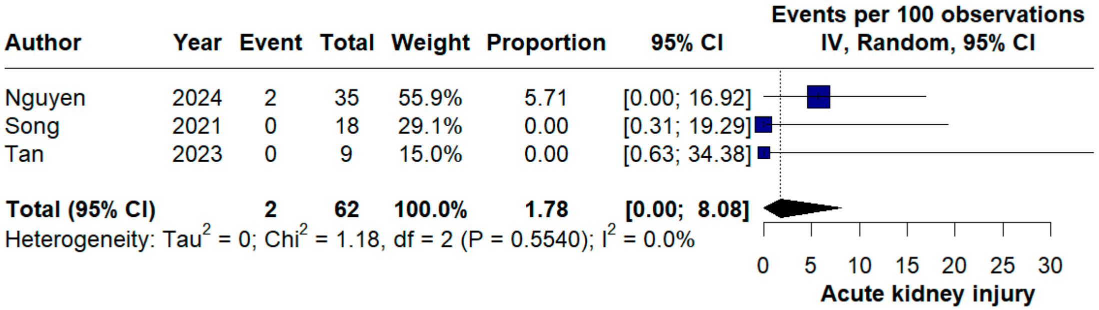

< 65 years

≥ 65 years

Author	Year	Event	Total	Weight	Proportion	95% CI
Cresce	2020	1	125	22.3%	0.80	[0.09; 4.52]
Hosoba	2023	0	216	26.7%	0.00	[0.02; 1.79]
Ito	2023	1	104	20.7%	0.96	[0.05; 5.31]
Yilmaz	2023	12	392	30.3%	3.06	[1.28; 4.98]
**Total (95% CI)**		**14**	**837**	**100.0%**	**0.96**	**[0.01; 2.86]**

Heterogeneity: $Tau^2 = 0.0031$; $Chi^2 = 11.96$, df = 3 (P = 0.0075); $I^2 = 74.9\%$

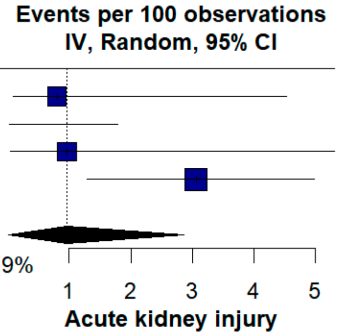

Figure S7. Subgroup analysis of acute kidney injury after TE-AVR according to mean patient age < 65 years [2,4,12] vs ≥ 65 years [3,7,10,11].

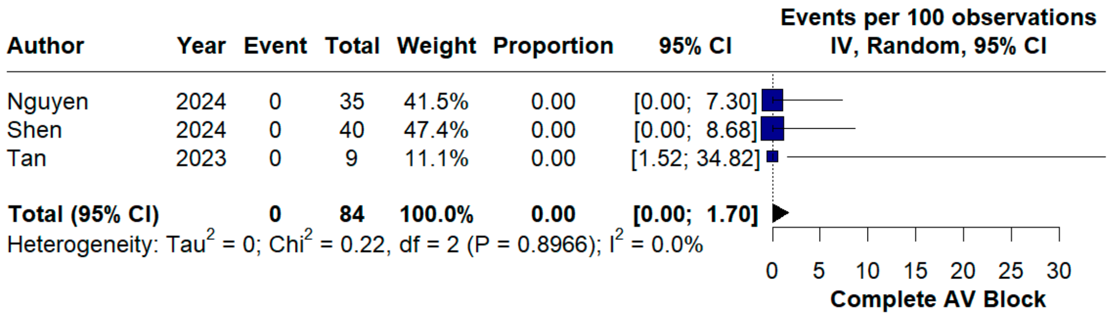

Figure S8. Subgroup analysis of complete atrio-ventricular block after TE-AVR in patients with a mean age < 65 years [2,4,6].

< 65 years

Author	Year	Event	Total	Weight	Proportion	95% CI
Gu	2023	2	29	22.3%	6.90	[0.00; 20.93]
Nguyen	2024	0	35	26.1%	0.00	[0.00; 10.00]
Shen	2024	0	40	29.1%	0.00	[0.00; 8.46]
Song	2021	0	18	14.7%	0.00	[0.40; 19.39]
Tan	2023	0	9	7.9%	0.00	[0.63; 34.38]
**Total (95% CI)**		**2**	**131**	**100.0%**	**0.27**	**[0.00; 3.24]**

Heterogeneity: $Tau^2 = 0.0014$; $Chi^2 = 3.83$, df = 4 (P = 0.4297); $I^2 = 0.0\%$

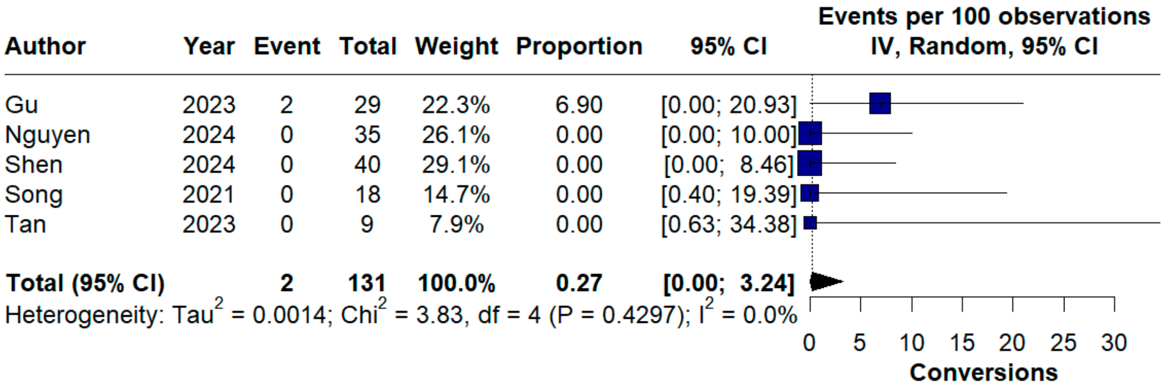

≥ 65 years

Author	Year	Event	Total	Weight	Proportion	95% CI
Cresce	2020	0	125	15.0%	0.00	[0.04; 3.06]
Hosoba	2023	2	216	25.8%	0.93	[0.18; 3.40]
Ito	2023	0	104	12.5%	0.00	[0.01; 3.55]
Yilmaz	2023	1	392	46.8%	0.26	[0.00; 1.10]
**Total (95% CI)**		**3**	**837**	**100.0%**	**0.22**	**[0.00; 0.77]**

Heterogeneity: $Tau^2 = 0$; $Chi^2 = 1.82$, df = 3 (P = 0.6104); $I^2 = 0.0\%$

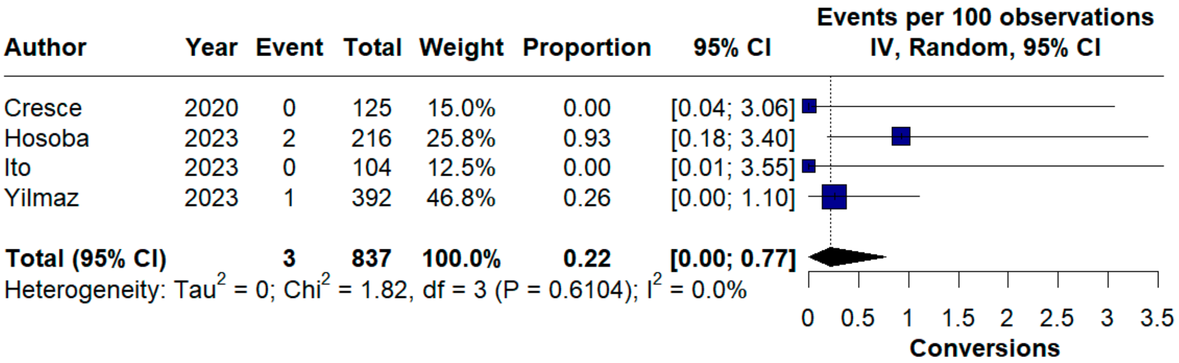

Figure S9. Subgroup analysis of conversion rates after TE-AVR according to mean patient age < 65 years [2,4–6,12] vs ≥ 65 years [3,7,10,11].

< 65 years

Author	Year	Event	Total	Weight	Proportion	95% CI
Nguyen	2024	1	35	40.5%	2.86	[0.00; 12.43]
Shen	2024	5	40	43.6%	12.50	[4.05; 26.73]
Tan	2023	0	9	15.9%	0.00	[1.52; 34.82]
**Total (95% CI)**		**6**	**84**	**100.0%**	**5.05**	**[0.11; 14.10]**

Heterogeneity: $Tau^2 = 0.0053$; $Chi^2 = 2.84$, df = 2 (P = 0.2419); $I^2 = 29.5\%$

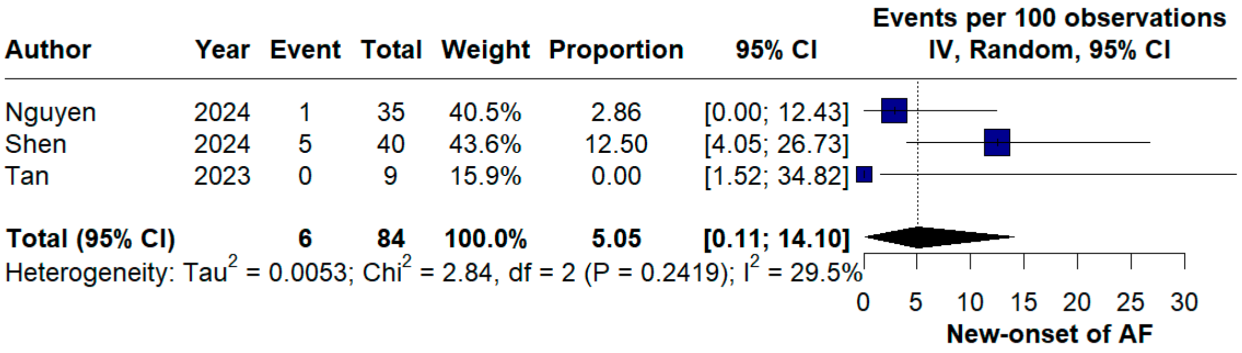

≥ 65 years

Author	Year	Event	Total	Weight	Proportion	95% CI
Cresce	2020	27	125	20.1%	21.60	[14.86; 29.92]
Hosoba	2023	16	216	20.6%	7.41	[4.39; 11.83]
Ito	2023	27	104	19.9%	25.96	[17.50; 35.32]
Tokoro	2020	0	47	18.5%	0.00	[0.20; 8.08]
Yilmaz	2023	97	392	20.9%	24.74	[20.34; 29.18]
**Total (95% CI)**		**167**	**884**	**100.0%**	**13.75**	**[4.16; 27.45]**

Heterogeneity: $Tau^2 = 0.0350$; $Chi^2 = 63.36$, $df = 4$ ($P < 0.0001$); $I^2 = 93.7\%$

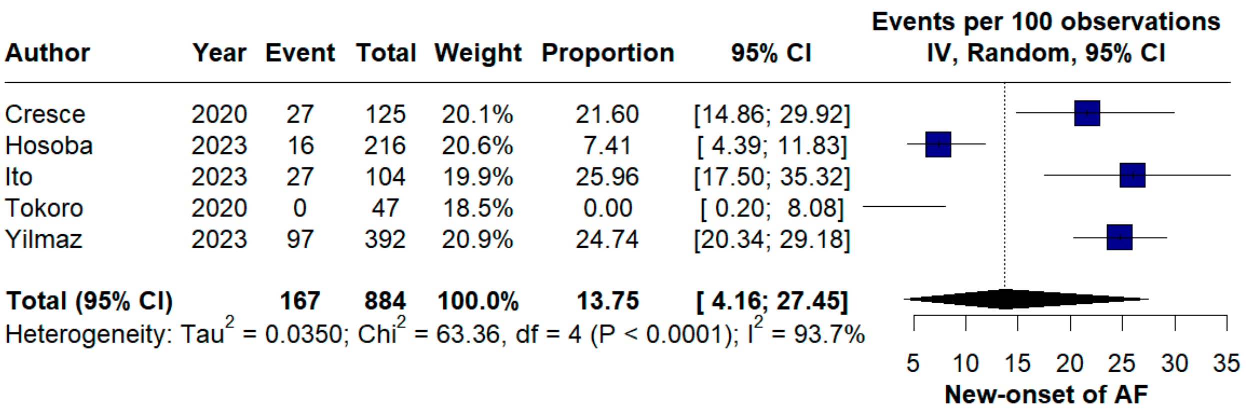

Figure S10. Subgroup analysis of new-onset atrial fibrillation after TE-AVR according to mean patient age < 65 years [2,4,6] vs ≥ 65 years [3,7,8,10,11].

< 65 years

Author	Year	Event	Total	Weight	Proportion	95% CI	Events per 100 observations IV, Random, 95% CI
Gu	2023	0	29	22.1%	0.00	[0.00; 9.53]	
Nguyen	2024	1	35	26.6%	2.86	[0.07; 14.92]	
Shen	2024	0	40	30.3%	0.00	[0.00; 8.46]	
Song	2021	0	18	13.9%	0.00	[0.40; 19.39]	
Tan	2023	0	9	7.1%	0.00	[0.63; 34.38]	
**Total (95% CI)**		**1**	**131**	**100.0%**	**0.08**	**[0.00; 2.37]**	

Heterogeneity: $Tau^2 = 0$; $Chi^2 = 1.39$, df = 4 (P = 0.8452); $I^2 = 0.0\%$

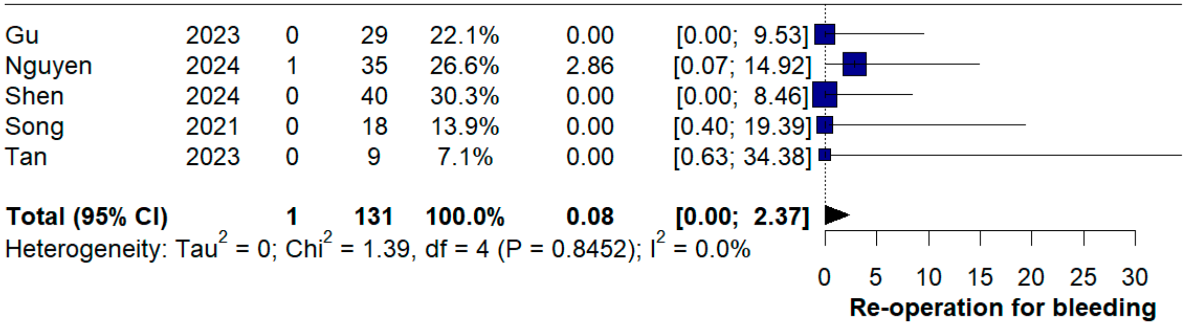

≥ 65 years

Author	Year	Event	Total	Weight	Proportion	95% CI	Events per 100 observations IV, Random, 95% CI
Cresce	2020	1	125	24.0%	0.80	[0.09; 4.52]	
Hosoba	2023	7	216	28.7%	3.24	[1.41; 6.65]	
Tokoro	2020	2	47	14.5%	4.26	[0.52; 14.54]	
Yilmaz	2023	25	392	32.8%	6.38	[3.93; 9.05]	
**Total (95% CI)**		**35**	**780**	**100.0%**	**3.43**	**[1.25; 6.49]**	

Heterogeneity: $Tau^2 = 0.0030$; $Chi^2 = 9.31$, df = 3 (P = 0.0254); $I^2 = 67.8\%$

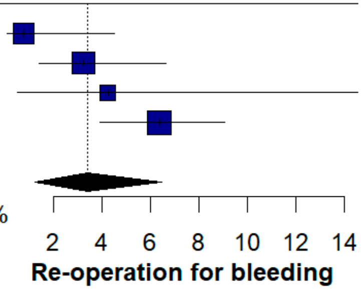

Figure S11.Subgroup analysis of re-operation for bleeding after totally endoscopic AVR according to mean patient age < 65 years [2,4–6,12] vs ≥ 65 years [3,7,8,10].

< 65 years

Author	Year	Event	Total	Weight	Proportion	95% CI
Gu	2023	0	29	11.9%	0.00	[0.00; 9.53]
Lin	2023	5	120	46.3%	4.17	[0.65; 8.68]
Nguyen	2024	0	35	14.2%	0.00	[0.00; 9.79]
Shen	2024	2	40	16.2%	5.00	[0.61; 16.92]
Song	2021	0	18	7.5%	0.00	[0.96; 19.87]
Tan	2023	0	9	3.9%	0.00	[0.63; 34.38]
**Total (95% CI)**		**7**	**251**	**100.0%**	**1.42**	**[0.05; 3.90]**

Heterogeneity: $Tau^2 = 0.0001$; $Chi^2 = 3.88$, df = 5 (P = 0.5662); $I^2 = 0.0\%$

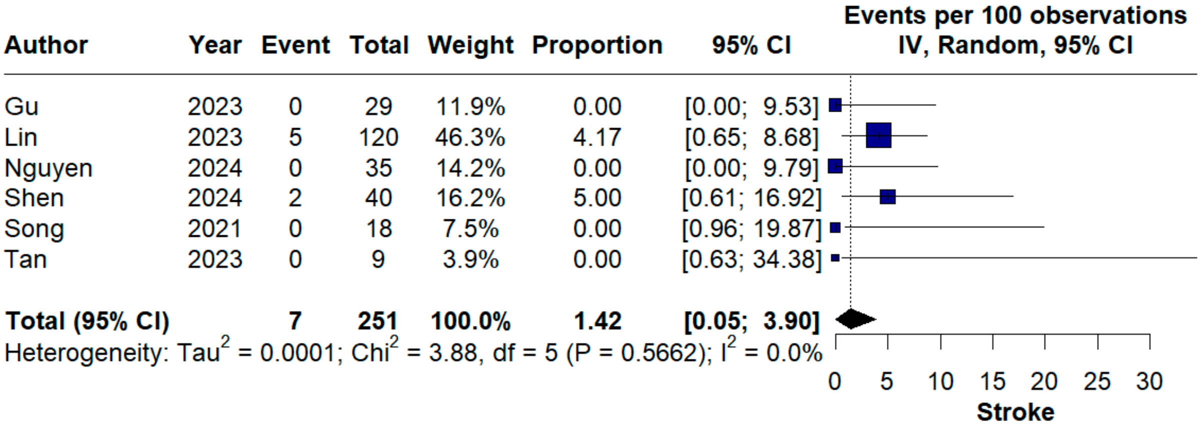

≥ 65 years

Author	Year	Event	Total	Weight	Proportion	95% CI
Cresce	2020	0	125	19.6%	0.00	[0.04; 3.06]
Hosoba	2023	2	216	23.6%	0.93	[0.18; 3.40]
Ito	2023	0	104	18.1%	0.00	[0.00; 3.00]
Tokoro	2020	3	47	11.8%	6.38	[1.87; 17.95]
Yilmaz	2023	8	392	27.0%	2.04	[0.59; 3.67]
**Total (95% CI)**		**13**	**884**	**100.0%**	**0.90**	**[0.01; 2.63]**

Heterogeneity: $Tau^2 = 0.0029$; $Chi^2 = 10.73$, df = 4 (P = 0.0298); $I^2 = 62.7\%$

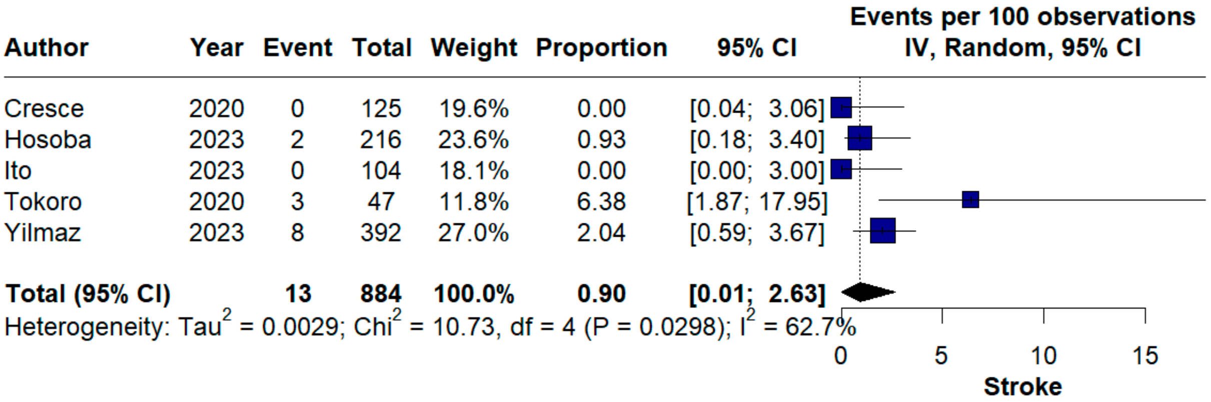

Figure S12. Subgroup analysis of early stroke after TE-AVR according to mean patient age < 65 years [2,4–6,9,12] vs ≥ 65 years [3,7,8,10,11].

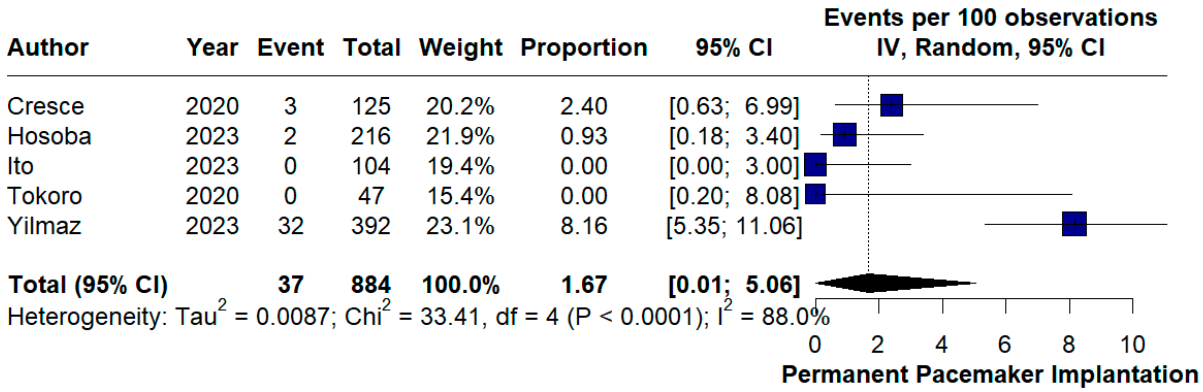

Figure S13. Subgroup analysis of permanent pacemaker implantation after TE-AVR in patients with a mean age of ≥ 65 years [3,7,8,10,11].

< 65 years

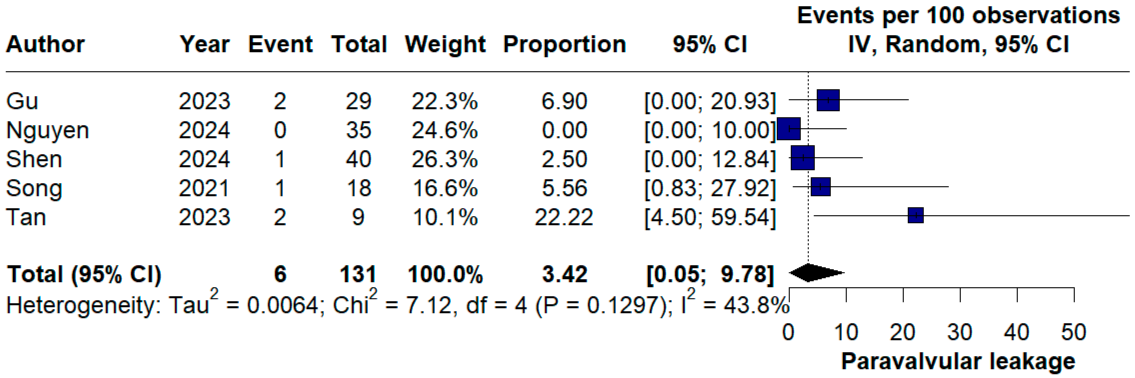

≥ 65 years

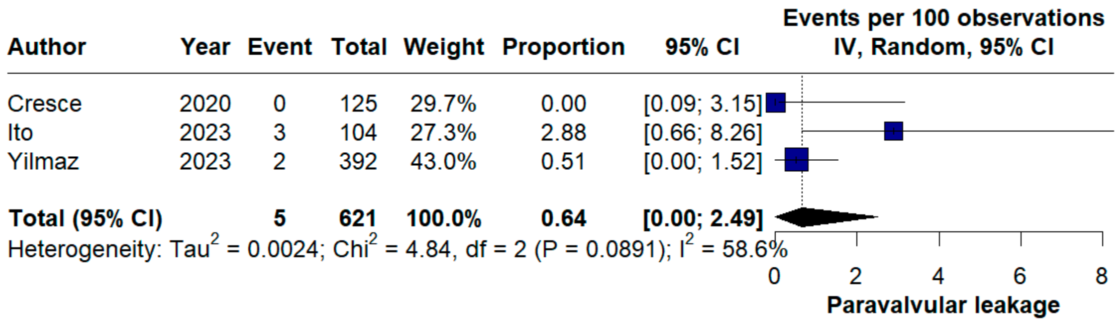

Figure S14. Subgroup analysis of paravalvular leakage after TE-AVR in patients with a mean age < 65 years [2,4–6,12] vs ≥ 65 years [7,10,11].

Author	Year	Total	Mean	SD	Weight	Mean	95% CI
Gu	2023	29	177.70	43.2000	16.5%	177.70	[161.98; 193.42]
Lin	2023	120	83.41	14.4000	17.1%	83.41	[80.83; 85.99]
Nguyen	2024	35	210.00	43.0000	16.6%	210.00	[195.75; 224.25]
Shen	2024	40	150.93	29.7100	16.9%	150.93	[141.72; 160.14]
Song	2021	18	128.90	28.2400	16.7%	128.90	[115.85; 141.95]
Tan	2023	9	137.56	28.0000	16.3%	137.56	[119.27; 155.85]
**Total (95% CI)**		**251**			**100.0%**	**147.78**	**[112.86; 182.70]**

Heterogeneity: $Tau^2 = 1859.5268$; $Chi^2 = 622.41$, df = 5 (P < 0.0001); $I^2 = 99.2\%$

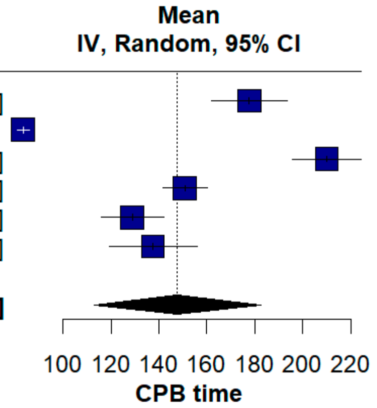

< 65 years **≥ 65 years**

Author	Year	Total	Mean	SD	Weight	Mean	95% CI
Cresce	2020	125	126.10	28.4000	20.3%	126.10	[121.12; 131.08]
Hosoba	2023	216	123.70	32.8000	20.4%	123.70	[119.33; 128.07]
Ito	2023	104	102.40	25.4000	20.4%	102.40	[97.52; 107.28]
Tokoro	2020	47	132.00	43.0000	18.2%	132.00	[119.71; 144.29]
Yilmaz	2023	392	93.23	23.6700	20.7%	93.23	[90.89; 95.57]
**Total (95% CI)**		**884**			**100.0%**	**115.05**	**[100.39; 129.70]**

Heterogeneity: $Tau^2 = 268.5084$; $Chi^2 = 255.04$, df = 4 (P < 0.0001); $I^2 = 98.4\%$

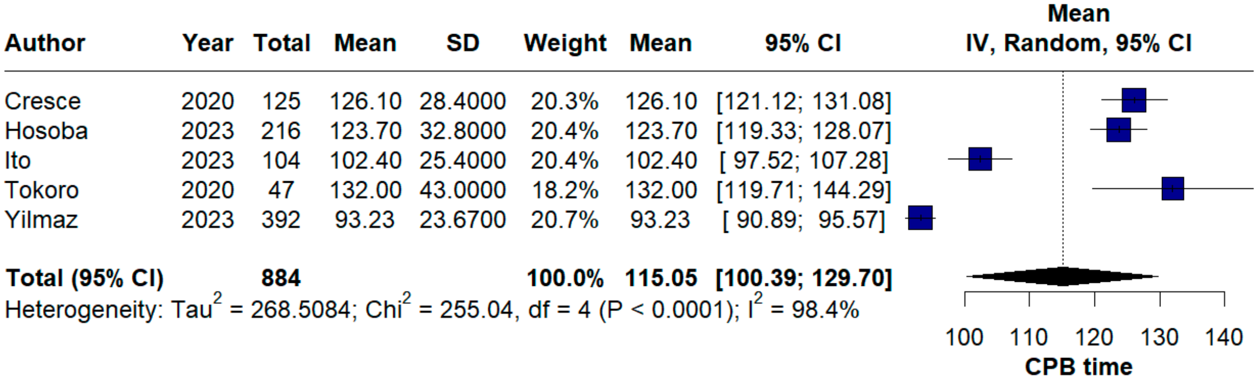

Figure S15. Subgroup analysis of cardiopulmonary bypass (CPB) time (min) in patients with a mean age < 65 years [2,4–6,9,12] vs ≥ 65 years [3,7,8,10,11].

< 65 years

Author	Year	Total	Mean	SD	Weight	Mean	95% CI
Gu	2023	29	2.30	1.1200	16.4%	2.30	[1.89; 2.71]
Lin	2023	120	1.35	0.7500	17.5%	1.35	[1.22; 1.48]
Nguyen	2024	35	3.00	0.0500	17.7%	3.00	[2.98; 3.02]
Shen	2024	40	1.20	0.4100	17.5%	1.20	[1.07; 1.33]
Song	2021	18	1.46	0.2700	17.6%	1.46	[1.34; 1.58]
Tan	2023	9	2.68	1.3000	13.3%	2.68	[1.83; 3.53]
**Total (95% CI)**		**251**			**100.0%**	**1.97**	**[1.35; 2.59]**

Heterogeneity: $Tau^2 = 0.5645$; $Chi^2 = 1854.92$, df = 5 (P = 0); $I^2 = 99.7\%$

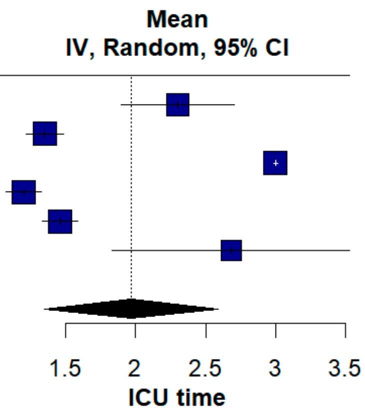

≥ 65 years

Author	Year	Total	Mean	SD	Weight	Mean	95% CI
Cresce	2020	125	1.91	2.4300	23.7%	1.91	[1.48; 2.34]
Hosoba	2023	216	1.35	0.7000	27.2%	1.35	[1.26; 1.44]
Tokoro	2020	47	1.20	0.0500	27.4%	1.20	[1.19; 1.21]
Yilmaz	2023	392	2.52	5.5600	21.7%	2.52	[1.97; 3.07]
**Total (95% CI)**		**780**			**100.0%**	**1.70**	**[1.13; 2.26]**

Heterogeneity: $Tau^2 = 0.3023$; $Chi^2 = 42.19$, df = 3 ($P < 0.0001$); $I^2 = 92.9\%$

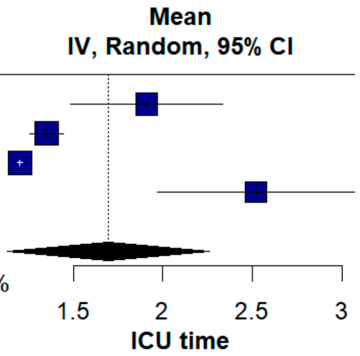

Figure S16. Subgroup analysis of intensive care unit (ICU) time (days) in patients with a mean age < 65 years [2,4–6,9,12] vs ≥ 65 years [3,7,8,10].

< 65 years

Author	Year	Total	Mean	SD	Weight	Mean	95% CI
Gu	2023	29	6.00	1.7000	24.9%	6.00	[5.38; 6.62]
Lin	2023	120	9.00	1.5000	25.2%	9.00	[8.73; 9.27]
Shen	2024	40	6.53	1.5800	25.0%	6.53	[6.04; 7.02]
Song	2021	18	11.78	1.2600	24.9%	11.78	[11.20; 12.36]
**Total (95% CI)**		**207**			**100.0%**	**8.33**	**[5.74; 10.92]**

Heterogeneity: $Tau^2 = 6.9026$; $Chi^2 = 259.64$, df = 3 (P < 0.0001); $I^2 = 98.8\%$

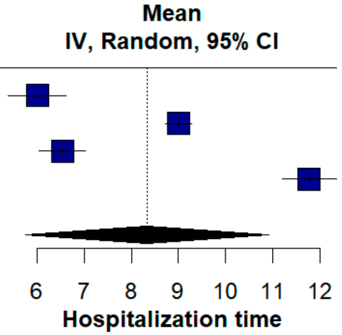

≥ 65 years

Author	Year	Total	Mean	SD	Weight	Mean	95% CI
Cresce	2020	125	8.30	9.3000	6.5%	8.30	[6.67; 9.93]
Hosoba	2023	216	8.00	1.5000	47.8%	8.00	[7.80; 8.20]
Ito	2023	104	7.57	9.7500	5.0%	7.57	[5.70; 9.44]
Tokoro	2020	47	7.40	2.0000	28.1%	7.40	[6.83; 7.97]
Yilmaz	2023	392	7.09	10.9600	12.7%	7.09	[6.01; 8.17]
**Total (95% CI)**		**884**			**100.0%**	**7.71**	**[7.27; 8.16]**

Heterogeneity: $Tau^2 = 0.0963$; $Chi^2 = 6.37$, df = 4 (P = 0.1729); $I^2 = 37.2\%$

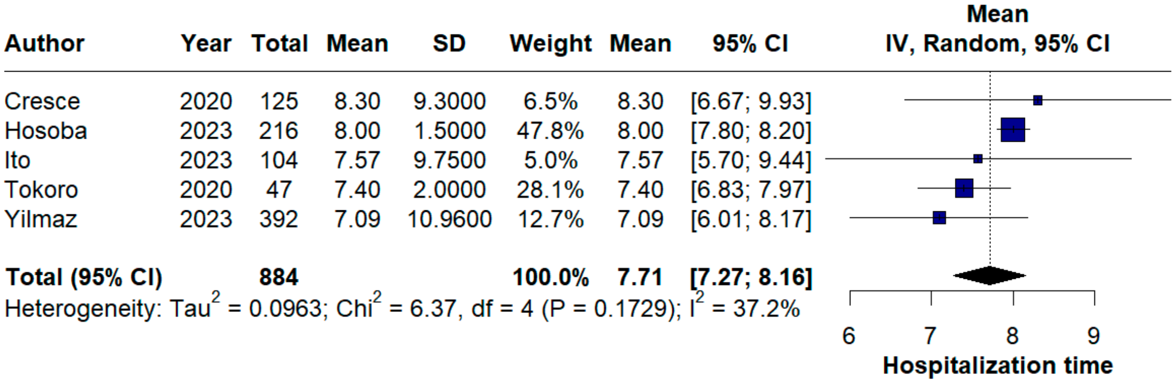

Figure S17. Subgroup analysis of hospitalization time (days) in patients with a mean age < 65 years [5,6,9,12] vs ≥ 65 years [3,7,8,10,11].

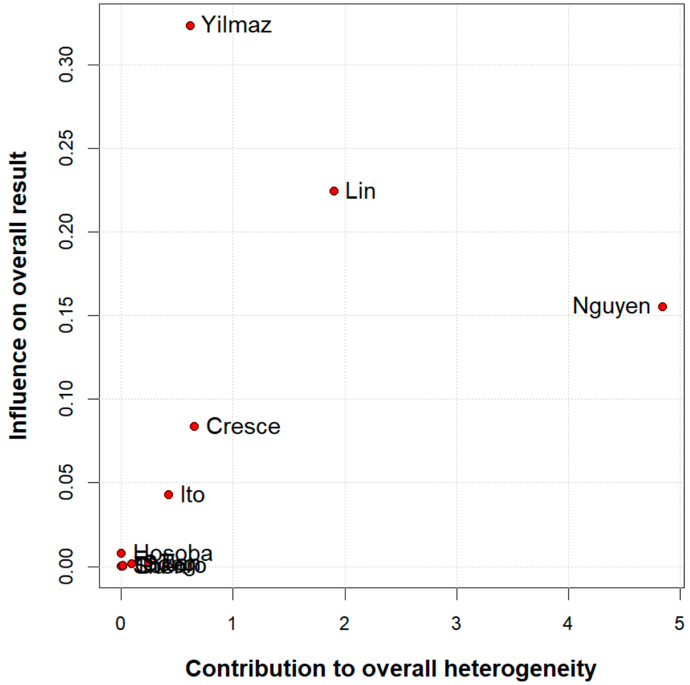

Figure S18. Baujat plot for hospital death [2–12].

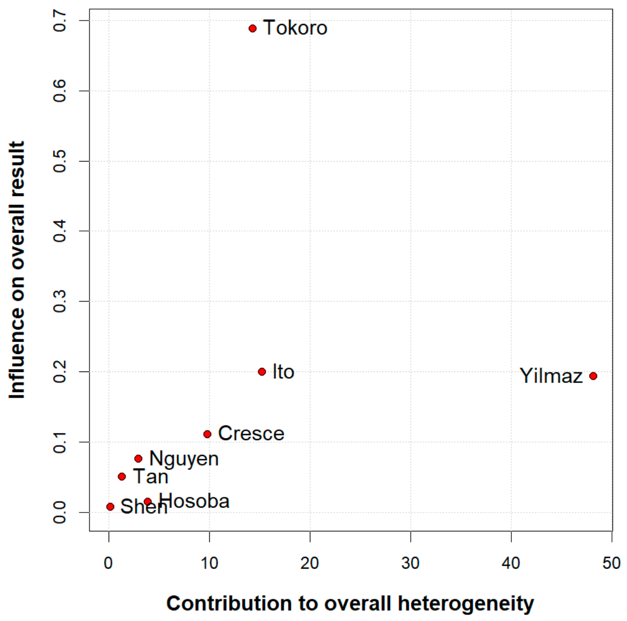

Figure S19. Baujat plot for New-onset atrial fibrillation [2–4,6–8,10,11].

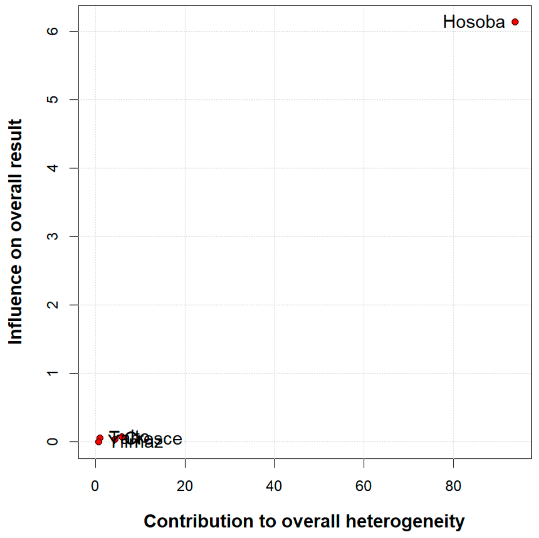

Figure S20. Baujat plot for Blood Transfusion [2,3,7,10,11].

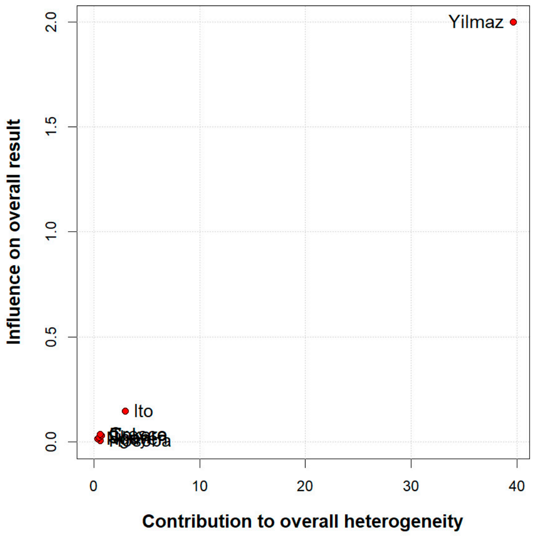

Figure S21. Baujat plot for Permanent Pacemaker Implantation [3,4,6–8,10,11].

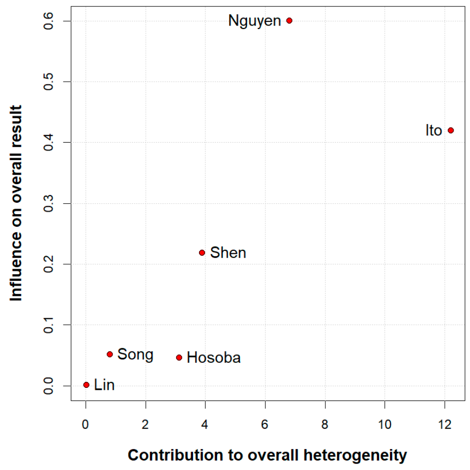

Figure S22. Baujat plot for Prolonged ventilation [3,4,6,9,11,12].

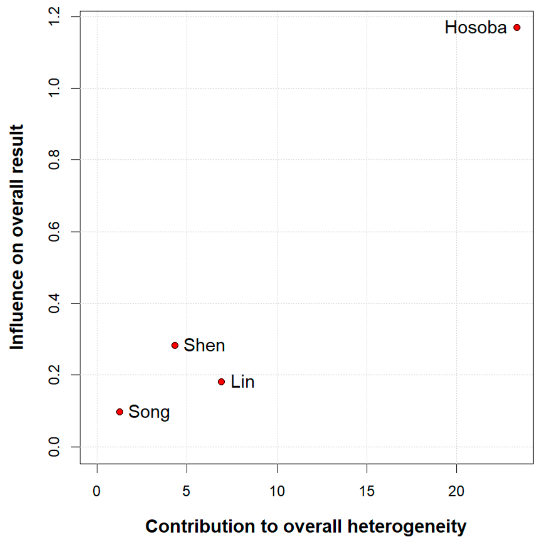

Figure S23. Baujat plot for Respiratory complications [3,6,9,12].

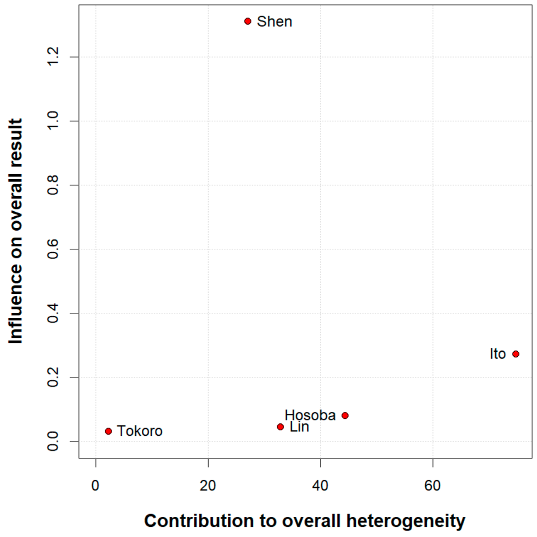

Figure S24. Baujat plot for Operation time [3,6,8,9,11].

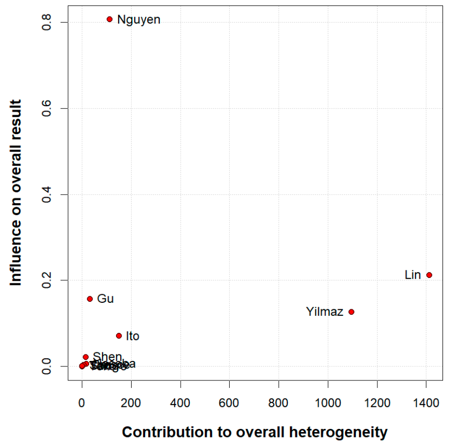

Figure S25. Baujat plot for CBP time [2–12].

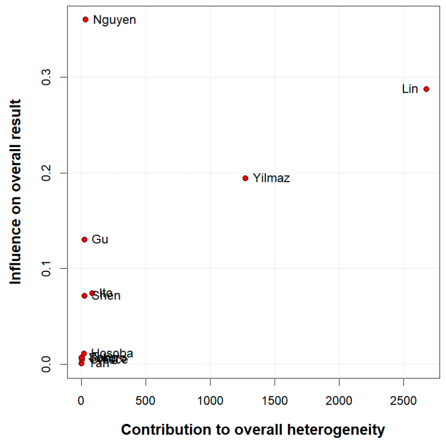

Figure S26. Baujat plot for ACC time [2–12].

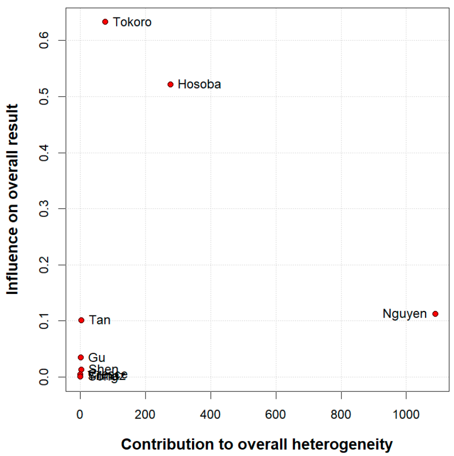

Figure S27. Baujat plot for Ventilation time [2–8,10,12].

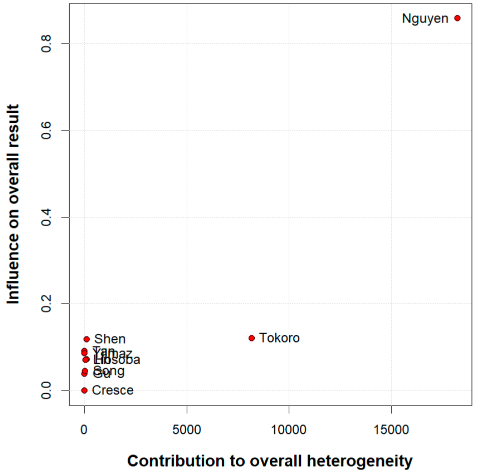

Figure S28. Baujat plot for ICU time [2–10,12].

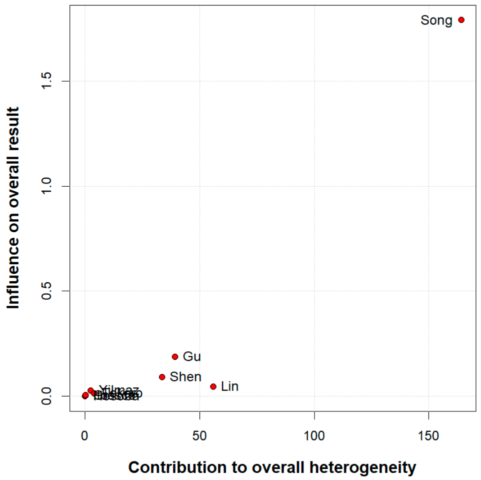

Figure S29. Baujat plot for Hospitalization time [3,5–12].

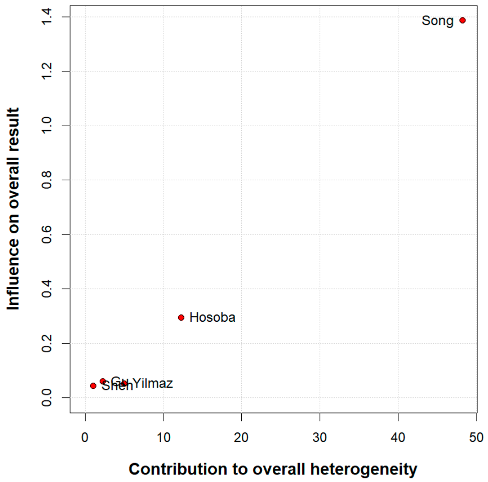

Figure S30. Baujat plot for Chest 24h-drainage [3,5–7,12].

Egger's test of the intercept

============================

intercept 95% CI t p

0.887 -0.22 - 1.99 1.573 0.1501655

Egger's test does not indicate the presence of funnel plot asymmetry.

Figure S31.Egger's test for hospitality death [2–12].

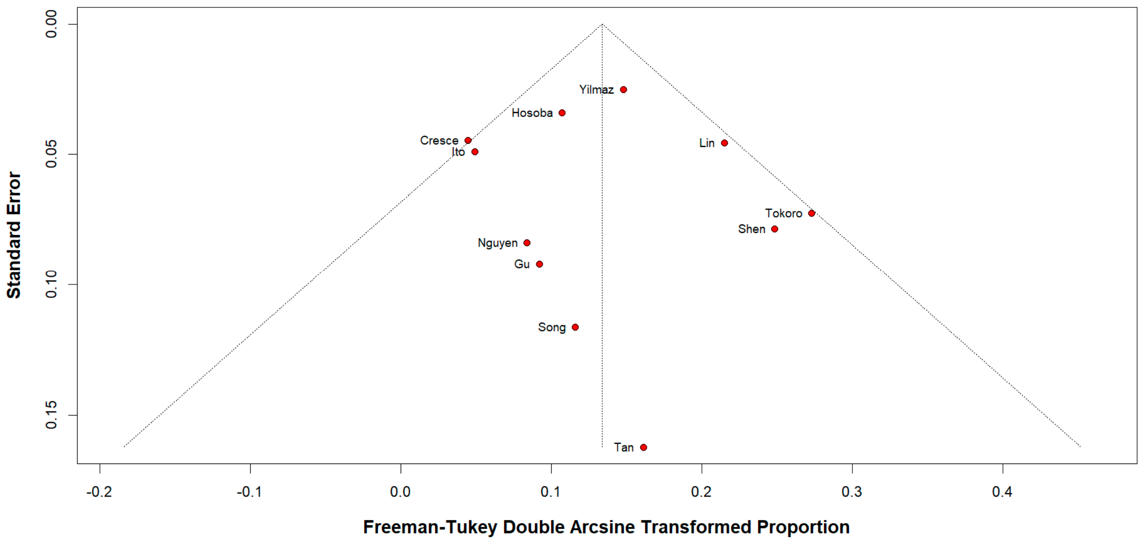

Egger’s test of the intercept

============================

intercept 95% CI t p

0.239 -1.51 - 1.99 0.268 0.7949443

Egger’s test does not indicate the presence of funnel plot asymmetry.

Figure S32. Funnel plot and Egger’s test for stroke [2–12].

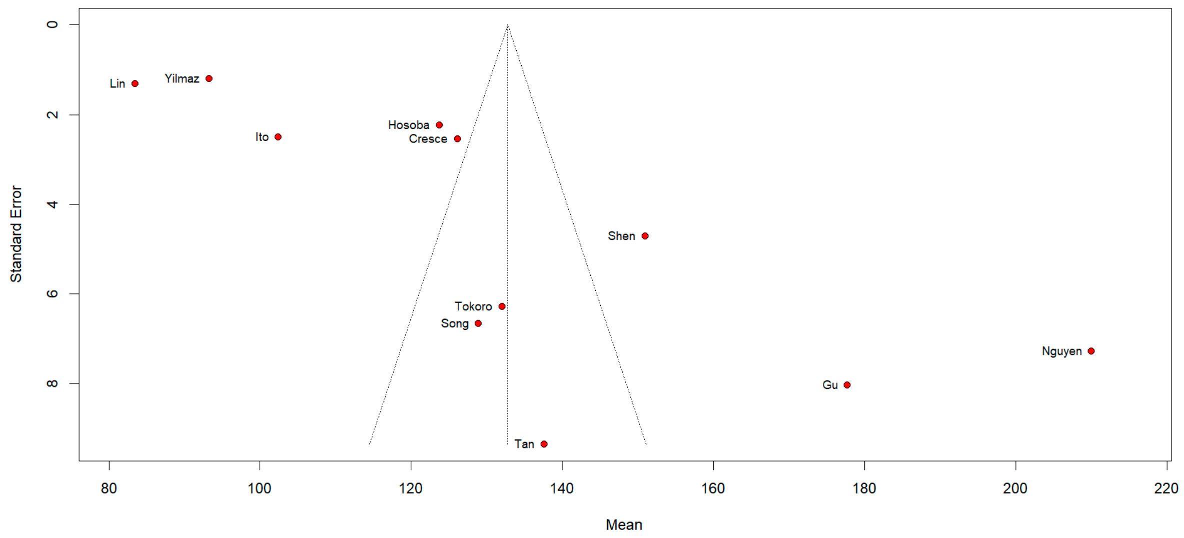

Egger’s test of the intercept

=============================

intercept 95% CI t p

12.681 6.89 - 18.47 4.293 0.002011611

Egger’s test indicates the presence of funnel plot asymmetry.

Figure S33. Funnel plot and Egger’s test for CPB time [2–12].

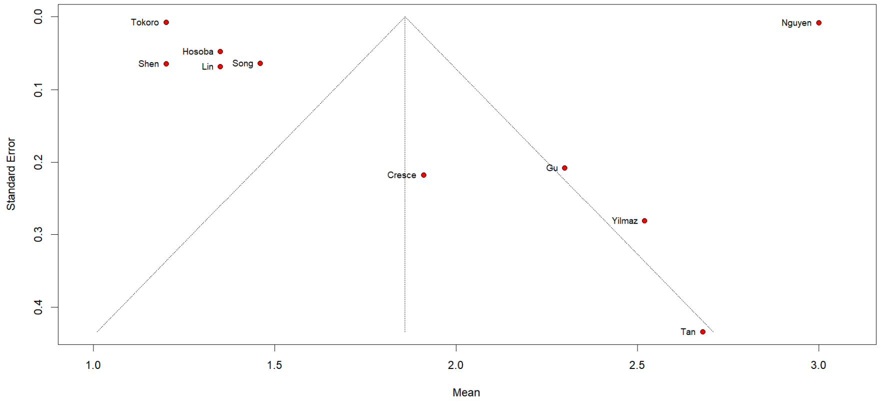

Egger’s test of the intercept

=============================

intercept 95% CI t p

-1.997 -45.67 - 41.68 -0.09 0.930787

Egger's test does not indicate the presence of funnel plot asymmetry

Figure S34. Funnel plot and Egger’s test for ICU time [2–10,12].

[1] Page MJ, McKenzie JE, Bossuyt PM, Boutron I, Hoffmann TC, Mulrow CD, et al. The PRISMA 2020 statement: an updated guideline for reporting systematic reviews. BMJ 2021:n71. https://doi.org/10.1136/bmj.n71.

[2] Tan T, Wei P, Liu Y, Huang H, Zhuang J, Chen J, et al. Safety and efficacy of two-port thoracoscopic aortic valve replacement. J Cardiothorac Surg 2023;18:9. https://doi.org/10.1186/s13019-022-02086-0.

[3] Hosoba S, Ito T, Mori M, Kato R, Kajiyama K, Maeda S, et al. Endoscopic Aortic Valve Replacement: Initial Outcomes of Isolated and Concomitant Surgery. Ann Thorac Surg 2023;116:744–9. https://doi.org/10.1016/j.athoracsur.2023.04.045.

[4] Nguyen HC, Pham DT. Totally 3D endoscopic aortic valve replacement: initial results and experience from a single center. Front Cardiovasc Med 2024;11:1468452. https://doi.org/10.3389/fcvm.2024.1468452.

[5] Gu W, Zhou K, Wang Z, Zang X, Guo H, Gao Q, et al. Totally endoscopic aortic valve replacement: Techniques and early results. Front Cardiovasc Med 2023;9:1106845. https://doi.org/10.3389/fcvm.2022.1106845.

[6] Shen H, Li D, Cheng N, Li L, Dong S, Shen H, et al. Comparative efficacy of totally thoracoscopic, mini-thoracotomy, and mini-sternotomy approaches in aortic valve replacement. Sci Rep 2024;14:17625. https://doi.org/10.1038/s41598-024-67018-5.

[7] Yilmaz A, Claessens J, Packlé L, Van Genechten S, Dönmez K, Awouters C, et al. Aortic Valve Replacement: Totally Endoscopic versus Mini-Sternotomy. J Clin Med 2023;12:7300. https://doi.org/10.3390/jcm12237300.

[8] Tokoro M, Sawaki S, Ozeki T, Orii M, Usui A, Ito T. Totally endoscopic aortic valve replacement via an anterolateral approach using a standard prosthesis. Interact Cardiovasc Thorac Surg 2020;30:424–30. https://doi.org/10.1093/icvts/ivz287.

[9] Lin Z, Chen X, Xu Z, Chen L, Dai X. Comparison of post-operative pain and quality of life between total thoracoscopic surgery and conventional full-sternotomy for aortic valve replacement. BMC Cardiovasc Disord 2023;23:580. https://doi.org/10.1186/s12872-023-03617-w.

[10] Cresce GD, Sella M, Hinna Danesi T, Favaro A, Salvador L. Minimally Invasive Endoscopic Aortic Valve Replacement: Operative Results. Semin Thorac Cardiovasc Surg 2020;32:416–23. https://doi.org/10.1053/j.semtcvs.2020.01.002.

[11] Ito J, Nakanaga H, Fujii H, Tabata M. Endoscopically assisted selective antegrade cardioplegia in minimally invasive aortic valve replacement for patients with aortic insufficiency. JTCVS Tech 2023;18:28–36. https://doi.org/10.1016/j.xjtc.2023.01.002.

[12] Song C, Jiang S, Zhu S, Fan Y. Early clinical outcomes of total thoracoscopic aortic valve replacement 2021. https://doi.org/10.22541/au.163253761.17854018/v1.
